# CMTM6 mediates the Warburg effect and promotes the liver metastasis of colorectal cancer

**DOI:** 10.1038/s12276-024-01303-1

**Published:** 2024-09-02

**Authors:** Aurpita Shaha, Yuanguo Wang, Xianghu Wang, Dong Wang, David Guinovart, Bin Liu, Ningling Kang

**Affiliations:** 1 Tumor Microenvironment and Metastasis, the Hormel Institute, University of Minnesota, Austin, MN USA; 2 Transcription and Gene Regulation, the Hormel Institute, University of Minnesota, Austin, MN USA; 3 Mathematical, Computational, and Statistical Modeling, the Hormel Institute, University of Minnesota, Austin, MN USA; 4 Present Address: The School of Medicine, Taizhou University, Taizhou, Zhejiang China

**Keywords:** Membrane trafficking, Metastasis

## Abstract

Liver metastasis of colorectal cancer (CRC) is a leading cause of death among cancer patients. The overexpression of glucose transporter 1 (Glut1) and enhanced glucose uptake that are associated with the Warburg effect are frequently observed in CRC liver metastases, but the underlying mechanisms remain poorly understood. CKLF-like MARVEL transmembrane domain-containing protein 6 (CMTM6) regulates the intracellular trafficking of programmed death-ligand-1 (PD-L1); therefore, we investigated whether CMTM6 regulates Glut1 trafficking and the Warburg effect in CRC cells. We found that knocking down of CMTM6 by shRNA induced the lysosomal degradation of Glut1, decreased glucose uptake and glycolysis in CRC cells, and suppressed subcutaneous CRC growth in nude mice and liver metastasis in C57BL/6 mice. Mechanistically, CMTM6 forms a complex with Glut1 and Rab11 in the endosomes of CRC cells, and this complex is required for the Rab11-dependent transport of Glut1 to the plasma membrane and for the protection of Glut1 from lysosomal degradation. Multiomics revealed global transcriptomic changes in CMTM6-knockdown CRC cells that affected the transcriptomes of adjacent cancer-associated fibroblasts from CRC liver metastases. As a result of these transcriptomic changes, CMTM6-knockdown CRC cells exhibited a defect in the G2-to-M phase transition, reduced secretion of 60 cytokines/chemokines, and inability to recruit cancer-associated fibroblasts to support an immunosuppressive CRC liver metastasis microenvironment. Analysis of TCGA data confirmed that CMTM6 expression was increased in CRC patients and that elevated CMTM6 expression was associated with worse patient survival. Together, our data suggest that CMTM6 plays multiple roles in regulating the Warburg effect, transcriptome, and liver metastasis of CRC.

## CMTM6: Key Player in CRC Metastasis and Tumor Microenvironment

Liver metastasis in colorectal cancer patients increases death rates, with current treatments often inadequate due to a lack of understanding of the underlying processes. This study explores how CRC cells change their metabolism to survive in the liver, focusing on the Warburg effect, where cancer cells use glycolysis preferentially. It focuses on the role of a protein called CMTM6 in this metabolic change. The researchers performed experiments on human and mouse CRC cells and used both in vitro and in vivo models, including mice with and without immune systems, to study the effects of CMTM6 on CRC growth and liver metastasis. The results showed reducing CMTM6 levels led to decreased glycolysis of CRC cells, CRC tumor growth and liver metastasis in mice. Future studies could lead to more effective treatments for CRC patients with liver metastases.

This summary was initially drafted using artificial intelligence, then revised and fact-checked by the author.

## Introduction

Liver metastasis of colorectal cancer (CRC) significantly contributes to the death of CRC patients, and further mechanistic insights and more effective treatment targets are needed for the treatment of CRC patients with liver metastases^1^. Metabolic reprogramming is a hallmark of cancer, as malignant cells must adapt their metabolic processes to produce the high amounts of energy that are required for their rapid growth, proliferation, and migration^2,3^. Among the numerous metabolic alterations, “the Warburg effect” is one of the best-known hallmarks of cancer; during the Warburg effect, cancer cells preferentially activate glycolytic pathways and produce lactate regardless of whether oxygen is present^4,5^. Due to the Warburg effect, positron emission tomography (PET) with [18 F] 2-fluoro-2-deoxy-D-glucose (FDG) has been widely used in the clinic to detect cancer^6^. The sensitivity of FDG PET in the detection of CRC liver metastases is approximately 97%^7^, suggesting that the Warburg effect is a metabolic target of CRC liver metastasis.

The first step in glycolysis is the uptake of glucose by cancer cells, which is facilitated by glucose transporter proteins on the plasma membrane (PM), such as glucose transporter 1 (Glut1). Overexpression of Glut1 has been observed in a variety of cancers, and high Glut1 expression is associated with poor prognosis and survival in CRC patients^8–10^. Both transcriptional and posttranscriptional mechanisms are involved in Glut1 expression in cancer cells. Similar to many other PM proteins, Glut1 undergoes trafficking between intracellular vesicular compartments and the PM. The small GTPase Rab11, which is a marker of recycling endosomes, facilitates the trafficking and recycling of PM proteins, including Glut1^11^, Glut2^12^, and Glut4^13^. We and others have demonstrated that in response to cytokine stimulation, Glut1 is targeted to the PM of cells via a Rab11-dependent mechanism^11,14,15^.

The CKLF-like MARVEL transmembrane domain (CMTM)-containing protein family includes eight members (CMTM1–8) that are involved in the trafficking of transmembrane proteins and secretory proteins^16^. It has been reported that CMTM6 protects programmed death-ligand-1 (PD-L1) from degradation by targeting it to the PM of cancer cells where it facilitates the immune evasion of cancer, including the immune evasion of CRC^17,18^. Consistently, increased CMTM6 expression has been observed in patients with pancreatic adenocarcinoma^19^, glioma^20^, head and neck squamous cell carcinoma^21^, and cervical cancer^22^. CMTM6 is also required for the maintenance of cancer stem cells and TGFβ-induced epithelial-to-mesenchymal transition (EMT) in cancer cells^21^. However, the role of CMTM6 in hepatocellular carcinoma is more complicated, as CMTM6 was shown to play both a tumor-suppressive role and a tumor-promoting role in hepatocellular carcinoma^23–25^.

Since CMTM6 is required for the intracellular trafficking of PD-L1 in CRC cells, in this study, we investigated whether CMTM6 regulates the trafficking and PM targeting of Glut1 to influence the Warburg effect and CRC liver metastasis. Our results revealed that shRNA-mediated CMTM6 knockdown effectively inhibited CRC tumor growth in immunocompromised mice and CRC liver metastasis in immunocompetent mice. In vitro, CMTM6 knockdown led to the lysosomal degradation of Glut1 as well as decreased glucose uptake and glycolysis in human and murine CRC cells. Mechanistically, CMTM6 was required for maintaining Rab11 mRNA levels, Rab11 activity, and Rab11-dependent transport of Glut1 to the PM. Targeted proteomics revealed that CMTM6 promoted the secretion of 60 cytokines/chemokines by CRC cells, which is important for shaping the tumor microenvironment. Multiomics revealed that targeting CMTM6 in CRC cells led to global transcriptomic changes in both CRC cells and cancer-associated fibroblasts (CAFs). Furthermore, analysis of TCGA data confirmed that CMTM6 expression was increased in CRC patients, and increased CMTM6 expression was associated with worse disease-free survival of CRC patients. Together, our data provide mechanistic insights into how CMTM6 regulates the Warburg effect, transcriptome, and liver metastasis of CRC.

## Materials and methods

### Cell lines and cell culture

The human CRC cell lines HCT116 and KM12L4 were purchased from ATCC (Manassas, VA, USA) and authenticated by short-tandem repeat DNA profiling by Genetica DNA Laboratories (Cincinnati, OH). MC38 mouse CRC cells were provided by Dr. Steven A. Rosenberg at NCI/NIH^26–28^. The cells were free of mycoplasma infection during the experiments. Cell proliferation was analyzed by the CellTiter 96® AQ_ueous_ One Solution Cell Proliferation Assay Kit (G3582 Promega, Madison, WI). Nunclon Sphera 96-well U-bottom ultra-low attachment cell culture plates were used to establish 3D tumoroids (174925 Thermo Fisher Scientific, Waltham, MA). Details about how cell plasma membrane integrity, the cell cycle and senescence, cell viability in tumoroids, and tumoroid compactness were determined are provided in the Supplementary Materials and Methods.

### Viral constructs and CRC cell transduction

Lentiviral constructs encoding CMTM6 shRNA were purchased from the Sigma MISSION shRNA library. Information about these constructs and the generation of the pMMPCMTM6-HA, pMMPRab11-FLAG, and pMMPRab11Q70L-FLAG retroviral vectors^15^ are provided in the Supplementary Materials and Methods. WB and sequencing were used to validate the constructs. Replication-defective lentiviruses or retroviruses were generated by transfecting 293 T/17 cells with 3 plasmids with the Effectene Transfection Reagent (301425 Qiagen, Tegelen Netherlands), as previously described^26,29,30^. Details about harvesting the viruses and transducing CRC cells with these viruses are provided in the Supplementary Materials and Methods.

### Protein detection and analysis

Glut1 on the PM was analyzed with a biotinylation assay, which included labeling the PM proteins with cell nonpermeable biotin EZ-Link Sulfo-NHS-Biotin (1 mg/mL in PBS, 21217 Thermo Fisher Scientific), pulling down the PM proteins with streptavidin agarose beads (S1638 Millipore Sigma), and performing Western blotting (WB) analysis with an anti-Glut1 antibody^26,31–33^. The Rab11 activity of CRC cells was evaluated by using a Rab11 activity assay kit (#83201 NewEast Biosciences, King of Prussia, PA)^15,34^. For coimmunoprecipitation (coIP), a buffer containing 0.5% NP-40 and a protease inhibitor cocktail (A32965 Thermo Fisher Scientific) was used to lyse the cells; 2 μg of anti-HA (12CA5) (11583816001 Roche, Millipore Sigma) or anti-FLAG (F1804 Millipore Sigma) antibody and 25 μL of Protein G Sepharose beads (50% slurry) (17061801 Cytiva, Marlborough, MA) were added to the cell lysates to pull down the protein complexes. Finally, the coprecipitated proteins were detected by WB^26,31,33,35^. WB and immunofluorescence (IF) staining were performed according to standard procedures^31–33^. Details are provided in the Supplementary Materials and Methods.

### In vitro protein binding

Recombinant Glut1-His and CMTM6-Strep proteins were expressed with the pFastBac baculovirus system. After the proteins were purified from the insect cells, an in vitro pull-down assay was performed to determine their in vitro binding using either an anti-His or anti-Strep antibody. The experimental details are provided in the Supplementary Materials and Methods.

### Glucose uptake and glycolysis assay

The fluorescent glucose analog 2-[*N*-(7-nitrobenz-2-oxa-1,3-diazol-4-yl) amino]-2-deoxyglucose (2-NBDG; Thermo Fisher Scientific) was used as a tracer for the glucose uptake assay, and its fluorescence in cells was measured by flow cytometry with a BD LSRFortessaTM X-20 Cell Analyzer and FlowJo_v10.8.1 software^15^. The glycolysis of CRC cells was studied with the Agilent Seahorse Glycolysis Stress Test with the Agilent Seahorse XFe96 Analyzer. The extracellular acidification rate (ECAR) in the cell culture medium was recorded in real time, and the data were analyzed by Seahorse Wave software^15^. Details are provided in the Supplementary Materials and Methods.

### RNA sequencing, spatial transcriptomics, and targeted proteomics

Bulk-cell RNA sequencing and spatial transcriptomics with the NanoString GeoMx Digital Spatial Profiler were performed by the University of Minnesota Genomic Center. In brief, tumor sections were prepared according to the Manual Slide Preparation User Manual (MAN-10150-01), fixed with formalin, and sent to the University of Minnesota Genomics Center for in situ hybridization, IF, area of interest (AOI) selection, sequencing, data acquisition and analysis^15^. The RNA sequencing data were analyzed by the EdgeR package^27,31,33^, and the spatial transcriptomic data were analyzed by GeoMx DSP Analysis Suite software; these data were subsequently used for GSEA and heatmap generation using online tools^15^. The cytokines/chemokines in the conditioned media of CRC cells were analyzed with a Proteome Profiler Mouse XL array kit (ARY028 R&D Systems, Minneapolis, MN, USA), which allows the simultaneous detection of 111 cytokines/chemokines. Chemiluminescence signals were captured with a ChemiDoc MP Imaging System (Bio-Rad, Hercules, CA), and the data were analyzed with ImageJ software (NIH).

### Subcutaneous tumor injection and portal vein tumor injection in mice

All the animal studies were approved by the Institutional Animal Care and Use Committee (IACUC) of the University of Minnesota. For subcutaneous tumor implantation, liver fibroblasts (0.75 × 10^6^)^26,31,32^ and human CRC cells (0.75 × 10^6^) were mixed in vitro and then subcutaneously co-injected into nude mice (553 Charles River Wilmington, MA). For portal vein tumor injection, two-month-old C57BL/6 mice were chosen as the recipients, and each mouse received 1 × 10^6^ CRC cells in 100 μL of PBS via portal vein injection under general anesthesia, as we previously described^26,27,33^.

### Statistical analysis

All the data are expressed as the means ± SEMs, and GraphPad Prism 6 software (GraphPad Software, Inc., La Jolla, CA) was used to perform the statistical analyses. For two group comparisons, two-tailed Student’s *t* tests were performed; for data from more than two groups, ANOVA followed by post hoc tests was performed. *P* < 0.05 was considered to indicate a significant difference.

## Results

### CMTM6 promotes CRC cell growth in 2D and 3D cultures

**Fig. 1:**
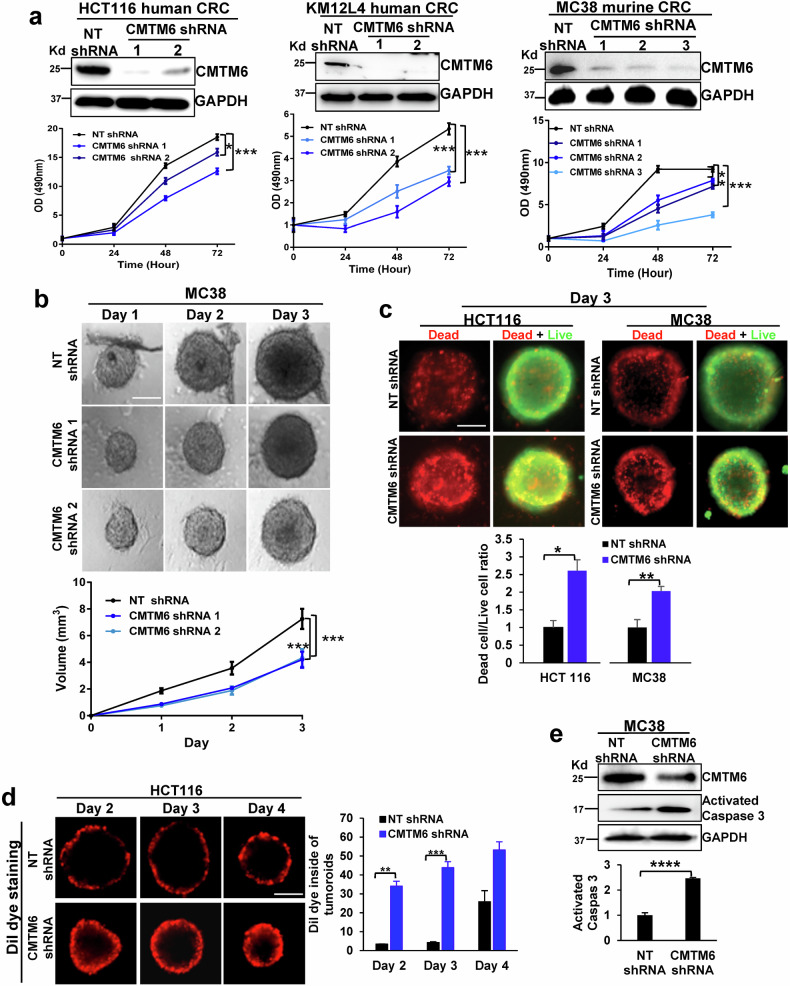
CMTM6 knockdown suppresses colorectal cancer cell (CRC) proliferation and tumoroid growth in vitro. **a** The proliferation of three CRC cell lines was measured by the CellTiter 96® AQ_ueous_ One Solution Cell Proliferation Assay. CMTM6 knockdown by different shRNAs consistently suppressed cell proliferation. *, *P* < 0.05; **, *P* < 0.01; ***, *P* < 0.001 according to *ANOVA*. *n* = 5. **b** MC38 cells were seeded in Nunclon Sphera 96-well U-bottom ultra-low attachment culture plates to induce 3D tumoroid formation. CMTM6 knockdown reduced the size of the tumor spheroids. ***, *P* < 0.001 according to *ANOVA*, *n* = 5. Scale bar, 1000 μm. **c** Live and dead cells in the tumoroids were analyzed with a LIVE/DEAD™ Viability/Cytotoxicity Kit (Invitrogen), and fluorescence microscopy was performed with an Axio observer (Zeiss). CMTM6 knockdown increased the dead/live ratio of the tumoroids. *, *P* < 0.05; **, *P* < 0.01 by *t* test, *n* = 5. Scale bar, 1000 μm. **d** The compactness of tumoroids was analyzed by Dil dye staining, and the Dil dye that penetrated the tumoroids was detected by an Axio observer. CMTM6-knockdown tumoroids were less compact than control tumoroids. **, *P* < 0.01; ***, *P* < 0.001 according to *ANOVA*, *n* = 3. Scale bar, 1000 μm. **e** Tumoroids were harvested and subjected to WB analysis of cleaved-caspase 3 (active) expression. CMTM6 knockdown increased active caspase 3 protein levels in the tumoroids. ****, *P* < 0.0001 by *t* test, *n* = 3.

To explore whether CMTM6 regulates CRC tumor growth via a mechanism independent of PD-L1-mediated cancer immune evasion, we generated control and CMTM6-knockdown CRC cells and compared their proliferation in vitro. HCT116 and KM12L4 human CRC cells were transduced with 2 different lentiviruses, each encoding a distinct CMTM6 shRNA, and cells transduced with nontargeting shRNA (NT shRNA) were used as controls. As revealed by the cell proliferation assay, knockdown of CMTM6 by 2 different shRNAs consistently suppressed the proliferation of both the HCT116 and KM12L4 human CRC cell lines (Fig. 1a left and middle panels, *P* < 0.05). In MC38 murine CRC cells, CMTM6 was knocked down by 3 different shRNAs, and the data were consistent with those of human CRC cells (Fig. 1a right panel, *P* < 0.05). Since 3D tumoroid culture better represents in vivo tumor growth than 2D cell culture, control and CMTM6-knockdown MC38 cells were subjected to 3D culture in ultra-low attachment cell culture plates (Nunclon Sphera 96-well U-bottom, Thermo Fisher Scientific). The results revealed that 3D tumoroid formation by MC38 cells was suppressed after CMTM6 knockdown (Fig. 1b, *P* < 0.001).

Next, the 3D tumoroids were analyzed with a Live-Dead Cell Staining Kit, and the dead cells in the tumoroids were labeled with red fluorescence while the live cells were labeled with green fluorescence. This assay revealed that the dead-to-live cell ratio was significantly higher in CMTM6-knockdown tumoroids than in control tumoroids (Fig. 1c, *P* < 0.05). Dil dye is normally excluded from compact tumoroids but can enter loosely formed tumoroids; therefore, it is used to assess the compactness of 3D tumoroids. As revealed by Dil dye staining, the compactness of CMTM6-knockdown tumoroids derived from either HCT116 or MC38 cells was compromised compared to that of control tumoroids (Fig. 1d and Supplementary Fig. 1a, *P* < 0.01). WB revealed that the level of cleaved-caspase 3 (activated-caspase 3) was higher in CMTM6-knockdown tumoroids than in control tumoroids (Fig. 1e, *P* < 0.0001). Propidium iodide (PI) is considered an indicator of cell plasma membrane integrity. PI staining of cultured live HCT116 cells confirmed that the percentage of PI-positive CRC cells was higher in CMTM6-knockdown cells than in control cells (Supplementary Fig. 1b, *P* < 0.001). Moreover, after long-term culture, CMTM6-knockdown HCT116 cells formed fewer and smaller colonies than control HCT116 cells in a colony formation assay (Supplementary Fig. 1c, *P* < 0.01). Thus, CMTM6 is important for CRC cell growth in vitro.

### Knockdown of CMTM6 leads to global transcriptomic changes in CRC cells and transcripts related to the cell cycle and glucose metabolism are affected

**Fig. 2:**
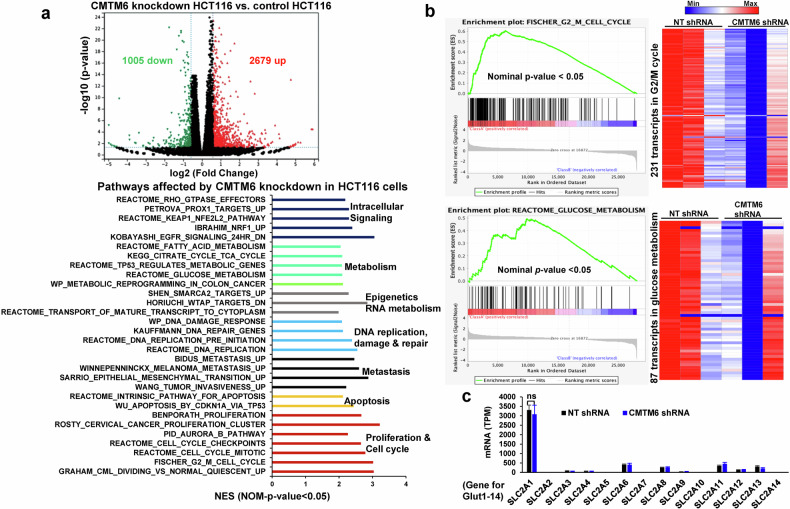
CMTM6 knockdown induces global transcriptomic changes in HCT116 CRC cells. **a** Upper panel, a volcano plot showing 1005 upregulated and 2679 downregulated transcripts after CMTM6 knockdown in HCT116 CRCs. The horizontal line represents *P* = 0.05. Vertical lines represent a fold change of 1.5. The green and red dots represent downregulated and upregulated genes, respectively. Lower panel, gene set enrichment analysis (GSEA) revealed numerous pathways that were affected by CMTM6 knockdown in HCT116 cells based on NES > 1 and *P* < 0.05. NES, normalized enrichment score. **b** Two gene sets whose gene expression was affected by CMTM6 knockdown are shown. Transcript enrichment is shown by an enrichment plot (left panel), and gene expression levels are shown by a heatmap (right panel). The scale bar represents the minimum (blue) to the maximum expression level (red). **c** mRNA levels of 14 different glucose transporters were analyzed by RNA sequencing. n.s., *P* > 0.05 by *t* test, *n* = 3.

To understand how CMTM6 knockdown suppressed CRC proliferation, we harvested control and CMTM6-knockdown HCT116 cells for bulk-cell RNA sequencing, and the data were analyzed with online software (http://sangerbox.com/home.html). A volcano plot showed that 1005 transcripts were downregulated and 2679 transcripts were upregulated by CMTM6 knockdown in HCT116 cells (Fig. 2a upper panel; the horizontal line represents *P* = 0.05, and the vertical lines represent a fold change of 1.5). According to the Gene Set Enrichment Analysis (GSEA) with molecular signatures database (C2 gene sets), the pathways and biological processes that were impacted by the transcriptomic changes included intracellular signaling, metabolism, epigenetics and RNA metabolism, DNA replication, damage and repair, metastasis, apoptosis, and cell cycle and proliferation (Fig. 2a lower panel, Nominal *P* < 0.05, NES > 1). For example, the levels of 231 transcripts that are involved the Fischer G2/M phase of the cell cycle and 87 transcripts that are involved in Reactome glucose metabolism were altered by CMTM6 knockdown in HCT116 cells (Fig. 2b).

These data led us to perform fluorescence-activated cell sorting (FACS) of PI-stained cells to determine whether CMTM6 knockdown influenced cell cycle progression, thereby affecting HCT116 cell proliferation and growth. Cell cycle analysis revealed that the percentage of control cells in the G2 phase of the cell cycle was 13.86%, whereas it was 30.19% in CMTM6-knockdown HCT116 cells (Supplementary Fig. 1d), suggesting that the G2-to-M phase transition of the cell cycle was indeed disrupted by CMTM6 knockdown. β-Galactosidase staining of senescent cells, however, did not reveal the induction of cell senescence by CMTM6 knockdown (Supplementary Fig. 1e, *P* > 0.05). Thus, CMTM6 knockdown in CRC cells results in global transcriptomic changes, and the G2-to-M phase transition is affected by these transcriptomic changes.

### CMTM6 knockdown suppresses CRC growth in immunocompromised mice

**Fig. 3:**
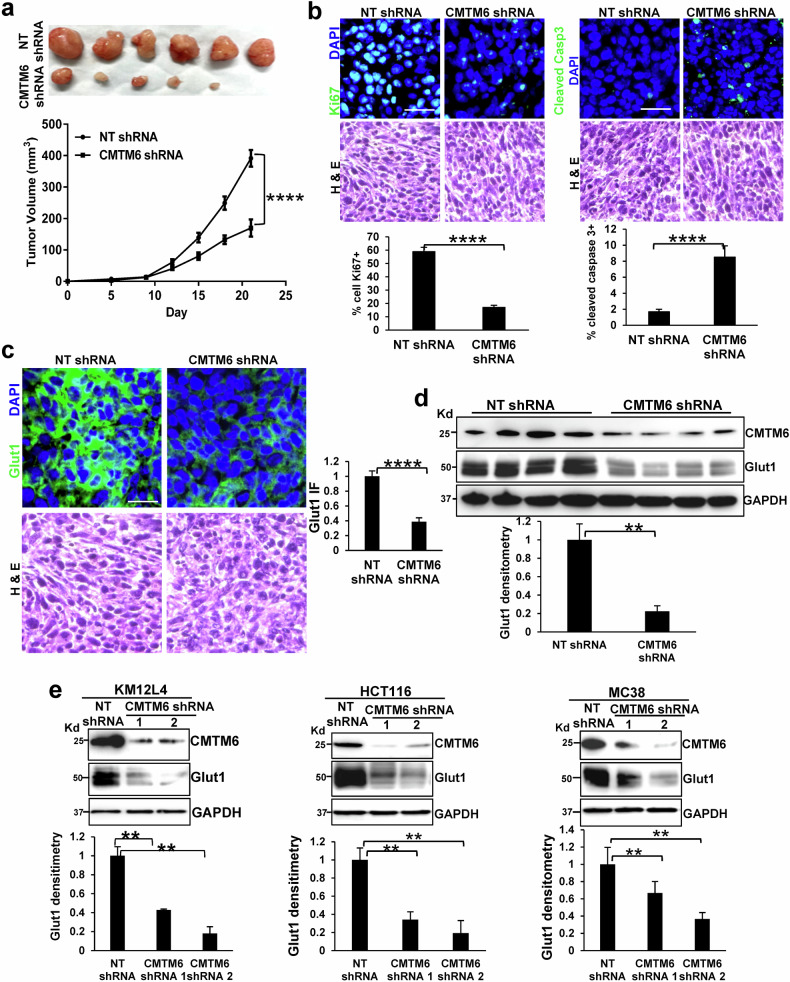
CMTM6 knockdown reduces CRC tumor growth in nude mice and the Glut1 protein level in CRC cells. **a** Control and CMTM6-knockdown HCT116 cells were mixed with liver fibroblasts, and they were subcutaneously coinjected into immunosuppressed nude mice. CMTM6 knockdown suppressed HCT116 tumor growth in nude mice. ****, *P* < 0.0001 according to ANOVA, *n* = 7,8. **b** Cryo-sections of HCT116 tumors were subjected to immunofluorescence (IF) staining for Ki67 (left) or cleaved caspase 3 (right). The cell nuclei were counterstained with DAPI (blue). The percentage of Ki67-positive cells was lower whereas the percentage of cleaved-caspase 3-positive cells was higher in CMTM6-knockdown tumors than in control tumors. ****, *P* < 0.0001 by *t test*, *n* = 7, 8. Scale bar, 50 μm. **c**, **d** IF and WB revealed reduced protein levels of glucose transporter 1 (Glut1) in CMTM6-knockdown tumors compared to control tumors. **, *P* < 0.01; ****, *P* < 0.0001 by *t* test, *n* = 4,4. Scale bar, 50 μm. **e** WB showed that Glut1 protein levels in CRC cells were reduced by CMTM6 knockdown in 2D culture. **, *P* < 0.01 according to *ANOVA*, *n* = 3.

The transcriptomic data and phenotypes of CMTM6-knockdown CRC cells led us to examine the role of CMTM6 in CRC growth in vivo. To minimize the influence of the immune system on CRC growth in mice, immunocompromised nude mice were selected as recipients for a tumor implantation study. Liver fibroblasts are among the most important components of the hepatic tumor microenvironment^36–38^. To determine the role of the liver tumor microenvironment in CRC growth and metastasis, we mixed liver fibroblasts (0.75 × 10^6^ cells)^26,34^ with control HCT116 cells (0.75 × 10^6^ cells) or CMTM6-knockdown HCT116 cells (0.75 × 10^6^ cells) in vitro and then coimplanted these cells into nude mice via subcutaneous injection. Tumor size was measured by a caliper on different days, and the data revealed that CMTM6 shRNA suppressed HCT116 tumor growth in mice (Fig. 3a, *P* < 0.0001). IF confirmed that the percentage of Ki67-positive cells was decreased, whereas that of active caspase 3-positive cells was increased, in CMTM6-knockdown HCT116 tumors compared to control HCT116 tumors (Fig. 3b, *P* < 0.0001). Since these mice are immunocompromised, these results suggest that CMTM6 can indeed promote CRC growth in mice through mechanisms that are independent of PD-L1-mediated immune evasion.

### CMTM6 knockdown reduces Glut1 protein levels, glucose uptake, and glycolysis in CRC cells

RNA sequencing identified glucose metabolism as one of the other pathways that are affected by CMTM6 knockdown. In addition, RNA sequencing revealed that among 14 glucose transporter isoforms, *SLC2A1* mRNA (encodes Glut1) was the predominant isoform that was expressed by HCT116 cells, whereas *SLC2A2* mRNA (encodes Glut2), *SLC2A3* mRNA (encodes Glut3), and *SLC2A4* mRNA (encodes Glut4) were barely detectable (Fig. 2c). Furthermore, no compensatory response of other glucose transporters to reduced Glut1 expression was observed in CMTM6-knockdown HCT116 cells (Fig. 2c). Since glucose uptake is the first step of glycolysis and increased Glut1 expression is associated with poor prognosis in cancers, including colon cancer^3,9,10^, WB and IF were subsequently performed to analyze Glut1 protein expression in control and CMTM6-knockdown tumors. As shown by IF and WB, Glut1 protein levels were lower in CMTM6-knockdown tumors than in control tumors (Fig. 3c, d, *P* < 0.01). Cultured HCT116, KM12L4, and MC38 cells were also collected and Glut1 expression was analyzed by WB; the results confirmed that Glut1 protein expression was drastically reduced by CMTM6 knockdown in 2D and 3D culture (Fig. 3e and Supplementary Fig. 2a, *P* < 0.01).

**Fig. 4:**
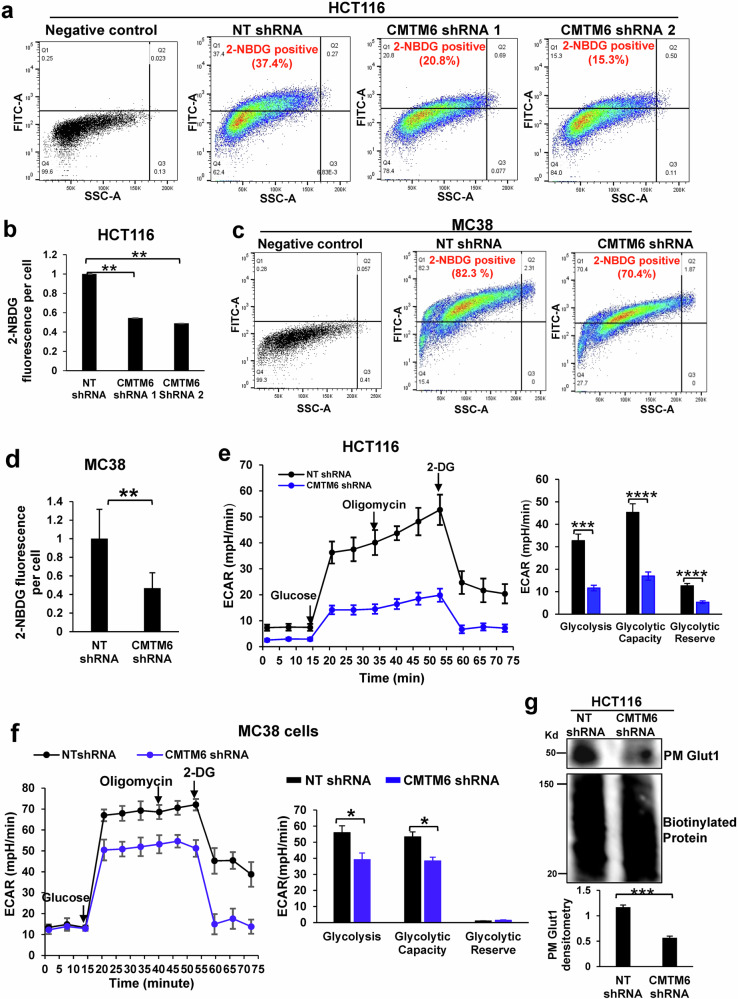
CMTM6 is required for the plasma membrane (PM) localization of Glut1, glucose uptake, and glycolysis in CRC cells. **a** A glucose uptake assay followed by flow cytometry analysis of 2-NBDG fluorescence demonstrated that the percentage of HCT116 cells that were positive for 2-NBDG uptake was reduced by CMTM6 knockdown. **b** The average 2-NBDG uptake by HCT116 cells was reduced by CMTM6 knockdown. **, *P* < 0.001 according to *ANOVA*; *n* > 5000 cells per group. **c** Glucose uptake assays showed that the percentage of MC38 cells that were positive for 2-NBDG uptake was reduced by CMTM6 knockdown. **d** The average 2-NBDG uptake by MC38 cells was reduced by CMTM6 knockdown. **, *P* < 0.01 by *t* test; *n* > 5000 cells per group. **e** The Agilent Seahorse Glycolysis Stress t**e**st was performed, and changes in the extracellular acidification rate (ECAR) were recorded in real time. Glycolysis in HCT116 cells was reduced by CMTM6 knockdown. ****, *P* < 0.0001 according to *ANOVA*, *n* = 5. **f** The Seahorse glycolysis stress test showed that glycolysis in MC38 cells was suppressed by CMTM6 knockdown. *, *P* < 0.05 according to *ANOVA*, *n* = 5. **g** A biotinylation assay revealed that the Glut1 level in the PM of HCT116 cells was reduced by CMTM6 knockdown. ***, *P* < 0.001 by *t* test, *n* = 3.

To determine whether CMTM6 knockdown influenced glucose uptake by CRC cells, CRC cells were incubated with the fluorescent glucose analog 2-(N-[7-nitrobenz-2-oxa-1,3-diazol-4-yl] amino)-2-deoxyglucose (2-NBDG), and flow cytometry was subsequently performed to quantify 2-NBDG uptake by the cells. The flow cytometry data confirmed that CMTM6 knockdown reduced 2-NBDG uptake by HCT116 and MC38 cells (Figs. 4a–d, *P* < 0.01). The Agilent Glycolysis Stress test measures changes in the extracellular acidification rate (ECAR) caused by the cellular secretion of lactate, which is indicative of real-time changes in glycolysis in cells^15^. The results of the Agilent Glycolysis Stress test showed that glycolysis was indeed suppressed by CMTM6 knockdown in the three CRC cell lines (Fig. 4e, f, and Supplementary Fig. 2b, *P* < 0.05). Since glucose transport is performed by Glut1 in the PM, we next performed a biotinylation assay and found that the Glut1 level in the PM was indeed significantly lower in CMTM6-knockdown HCT116 and MC38 cells than in control cells (Fig. 4g and Supplementary Fig. 2c, *P* < 0.01). Thus, CMTM6 is required for maintaining Glut1 levels, glucose uptake, and glycolysis in CRC cells.

### CMTM6 directly binds to Glut1, and its knockdown leads to lysosomal degradation of Glut1

**Fig. 5:**
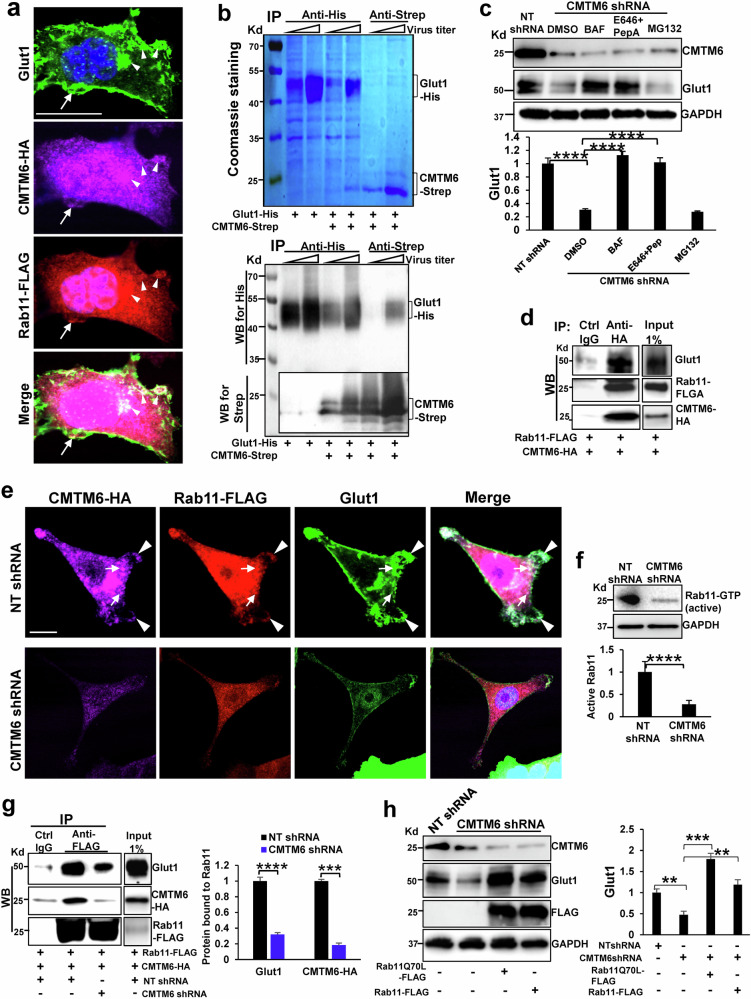
CMTM6 forms a complex with Glut1 and Rab11 that is required for the PM targeting of Glut1. **a** IF staining showed that Glut1 (green), CMTM6-HA (purple), and Rab11-FLAG (red) colocalized in the PM (arrows) and endosomes (arrowheads) of HCT116 cells. Scale bar, 20 µm. **b** Cultured insect cells were transduced with baculoviruses to express recombinant Glut1-His and CMTM6-Strep proteins. Cell membrane proteins were dissolved, purified, and subjected to pulldown. Coomassie staining (upper) and WB (lower panel) revealed that CMTM6-Strep was coprecipitated with Glut1-His by an anti-His antibody (Lanes 4, 5), and conversely, Glut1-His was coprecipitated with CMTM6-Strep by an anti-Strep antibody (Lanes 6, 7). **c** CMTM6-knockdown HCT116 cells were incubated with lysosomal inhibitor(s) [bafilomycin (BAF) or E64d + pepstatin A (PepA)] or the proteasomal inhibitor MG132, and the cells were collected for WB analysis. The lysosomal inhibitor(s), but not the proteasomal inhibitor, protected Glut1 from downregulation due to CMTM6 knockdown. ****, *P* < 0.0001 according to *ANOVA*, *n* = 3. **d** HCT116 cells expressing CMTM6-HA and Rab11-FLAG after retroviral transduction were collected for coimmunoprecipitation (coIP), and the results revealed that CMTM6 interacted with Glut1 and Rab11 in HCT116 cells. The data are representative of multiple repeats with similar results. **e** Representative triple IF images of CMTM6-HA (purple), Rab11-FLAG (red), and Glut1 (green) in control and CMTM6-knockdown HCT116 cells are shown. Scale bar, 10 µm. **f** Rab11 activity assays revealed that the Rab11-GTP level in HCT116 cells was reduced by CMTM6 knockdown. ****, *P* < 0.0001 by *t* test, *n* = 3. **g** HCT116 cells overexpressing CMTM6-HA and Rab11-FLAG were harvested for coIP. CMTM6 knockdown reduced the Glut1/Rab11 interaction in HCT116 cells. ***, *P* < 0.001; ****, *P* < 0.0001 according to *ANOVA*, *n* = 3. **h** WB showed that Glut1 in CMTM6 knockdown cells was restored by the expression of a constitutively active Rab11Q70L-FLAG mutant or wild-type Rab11-FLAG and that the Rab11Q70L-FLAG mutant exerted a stronger effect. **, *P* < 0.01; ***, *P* < 0.001 according to ANOVA, *n* = 3.

As shown in Fig. 2c, Glut1 mRNA levels in control and CMTM6-knockdown HCT116 cells were comparable (*P* = 0.71). Therefore, we investigated whether CMTM6 regulated Glut1 expression via a posttranscriptional mechanism similar to its regulation of PD-L1. To this end, HCT116 cells overexpressing CMTM6-HA and Rab11-FLAG were subjected to triple IF, and the results revealed that Glut1 (green) and CMTM6 (purple) colocalized with endosomes (arrowheads, Fig. 5a) and the PM (arrows, Fig. 5a) of CRC cells. Additionally, these proteins colocalized with Rab11, which is a marker of recycling endosomes (red) (Fig. 5a). To confirm the CMTM6/Glut1 interaction, 2 baculoviral vectors, one encoding His-tagged Glut1 and another encoding Strep-tagged CMTM6, were generated to produce recombinant proteins. His-tagged Glut1 and Strep-tagged CMTM6 were generated and purified from insect cells cotransduced with the 2 baculoviruses, and these proteins were used for pull-down assays to analyze the CMTM6/Glut1 interaction in vitro. Glut1-His and CMTM6-Strep were coprecipitated with either an anti-His or anti-Strep antibody, as revealed by Coomassie blue staining and WB (Fig. 5b, Lanes 4-7); these results indicated the direct interaction between the CMTM6 and Glut1 proteins.

Glut1 is degraded by lysosomes^15^, so we next investigated whether CMTM6 knockdown led to increased lysosomal targeting and degradation of Glut1. CMTM6-knockdown CRC cells were incubated with either lysosomal inhibitor(s) (bafilomycin or E646 + pepstatin A) or the proteasomal inhibitor MG132. Cells incubated with DMSO were used as controls. WB revealed that the CMTM6 shRNA-induced downregulation of Glut1 was abolished by the lysosomal inhibitor(s), bafilomycin or E646 + pepstatin A (Fig. 5c, *P* < 0.0001), but not by the proteasomal inhibitor MG132 (Fig. 5c); these results indicated that CMTM6 knockdown indeed led to lysosomal targeting and degradation of Glut1 in CRC cells.

### CMTM6 knockdown reduces Rab11 mRNA and protein levels, Rab11 activity, and the Rab11/Glut1 interaction

The transport of Glut1 from endosomes to the PM depends on Rab11^11,14,15^, which is a small GTPase that plays a key role in the recycling of cargo proteins to the PM^39–42^. Therefore, we collected CRC cells that coexpressed CMTM6-HA and Rab11-FLAG and performed coimmunoprecipitation (coIP) to analyze the CMTM6/Glut1/Rab11 interaction in CRC cells. An anti-HA antibody was used to pull down CMTM6-HA, followed by WB to detect the coprecipitated Glut1 and Rab11-FLAG. Rab11-FLAG and Glut1 were coprecipitated with CMTM6-HA by an anti-HA antibody, confirming that the three proteins formed a complex in CRC cells (Fig. 5d). Triple IF demonstrated the colocalization of the three proteins in endosomes (arrows) and in the PM (arrowheads) of control HCT116 cells (Fig. 5e). IF also demonstrated that CMTM6 knockdown downregulated not only Glut1 protein expression (Fig. 5e, green) but also Rab11 protein expression (Fig. 5e, red), and this finding was confirmed by WB (Supplementary Fig. 2d*P* < 0.0001). RNA sequencing data showed that the Rab11 mRNA level in CMTM6-knockdown cells was only half that in control cells (Supplementary Fig. 2e, *P* < 0.05). A Rab11 activity assay, which measures the level of active Rab11 (Rab11-GTP), revealed that Rab11 activity in CRC cells was suppressed by CMTM6 knockdown (Fig. 5f, *P* < 0.0001). Moreover, coIP demonstrated that CMTM6 knockdown disrupted the Rab11/Glut1 interaction in CRC cells (Fig. 5g, *P* < 0.0001). To further explore the role of Rab11 in the CMTM6 knockdown-induced downregulation of Glut1, a constitutively active Rab11 mutant construct, namely, Rab11Q70L^43^, was created by site-directed mutagenesis, and its role in rescuing Glut1 in CMTM6-knockdown cells was assessed. To this end, cells overexpressing the Rab11Q70L mutant or wild-type Rab11 after retroviral transduction were subjected to shRNA-mediated CMTM6 knockdown. WB data demonstrated that both were able to restore the Glut1 protein level in CMTM6-knockdown cells, but the Rab11Q70L mutant exerted a stronger effect (Fig. 5h, *P* < 0.05). Thus, the Glut1 downregulation that was observed in CMTM6-knockdown CRC cells was indeed mediated by Rab11.

### CMTM6 promotes CRC liver metastasis in a liver metastasis mouse model

**Fig. 6:**
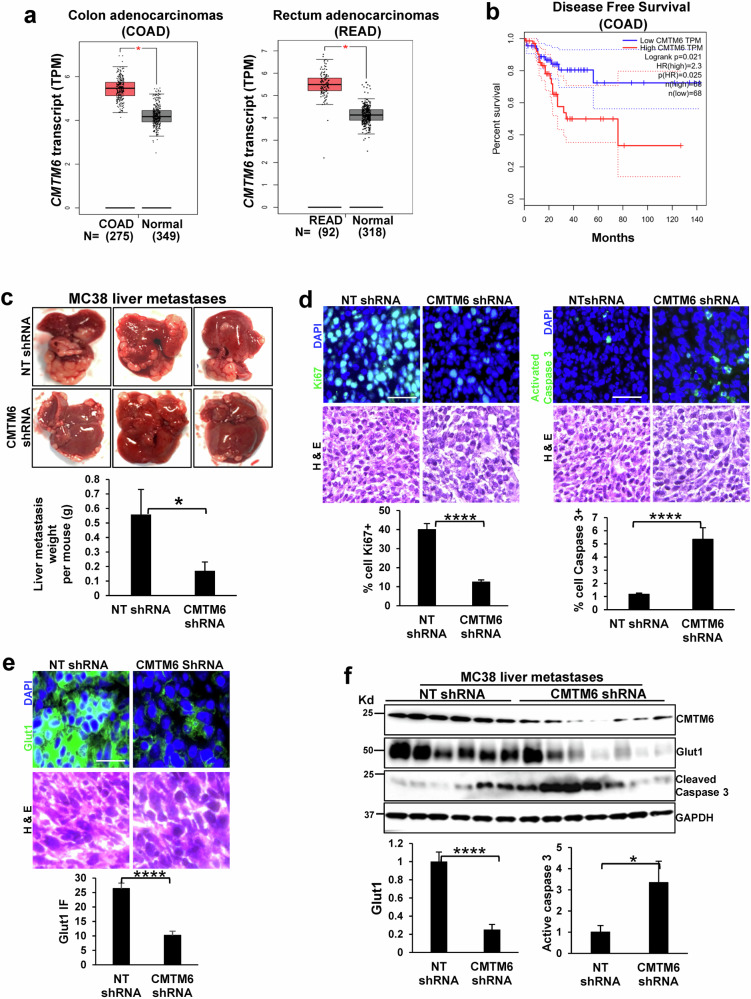
CMTM6 knockdown suppresses MC38 liver metastasis. **a** Patient data from the TCGA database showed that *CMTM6* expression is higher in adenocarcinoma of the colon and rectum than in normal tissues. *, *P* < 0.05 by *t* test. The number of patients (**N)** is shown. **b** Elevated CMTM6 expression in CRC tissues was associated with worse disease-free survival in CRC patients. *P* = 0.021 by log-rank test. *n* = 68 per group. **c** MC38 cells expressing NT shRNA (control) or CMTM6 shRNA were injected into mouse livers via the portal vein. CMTM6 knockdown suppressed MC38 cell-derived liver metastasis in the mice. *, *P* < 0.05 by *t* test, *n* = 6,7. **d** IF s*t*aining revealed that the percentage of Ki67-positive cells was reduced, whereas the percentage of activated-caspase 3-positive cells was increased, in CMTM6-knockdown liver metastases compared to control liver metastases. ****, *P* < 0.0001 by *t* test, *n* = 6,7. Scale bar, 50 μm. **e** IF staining revealed reduced Glut1 in CMTM6-knockdown liver metastases compared to control liver metastases. ****, *P* < 0.0001, *n* = 6,7 by *t* test. Scale bar, 25 μm. **f** WB showed that *t*he average Glut1 level was lower, whereas the average activated-caspase 3 level was higher in CMTM6-knockdown liver metastases than in control liver metastases. *, *P* < 0.05; ****, *P* < 0.0001 by *t* test, *n* = 6,7.

We next used patient RNA sequencing data from The Cancer Genome Atlas (TCGA) to analyze CMTM6 expression in CRC using a web server and Gene Expression Profiling Interactive Analysis. CMTM6 expression was higher in 275 colon adenocarcinoma (COAD) tissues than in 349 control tissues, and CMTM6 expression was also higher in 92 rectum adenocarcinoma (READ) tissues than in 318 control tissues (Fig. 6a, *P* < 0.05). Additionally, elevated CMTM6 expression was associated with worse disease-free survival in a cohort of COAD patients (Fig. 6b, *P* < 0.05). Since liver metastasis significantly contributes to the death of CRC patients, we next investigated whether CMTM6 promoted CRC liver metastasis in a portal vein tumor injection mouse model. MC38 cells expressing NT shRNA or CMTM6 shRNA were implanted into the livers of mice via portal vein injection (1 × 10^6^ cells per mouse), and all the mice were killed 11 days later. We found that MC38 cell-derived liver metastasis in mice was significantly suppressed by shRNA-mediated CMTM6 knockdown (Fig. 6c, *P* < 0.05). IF showed that the percentage of Ki67-positive cells was reduced, whereas that of active caspase 3-positive cells was increased, in CMTM6-knockdown MC38 cell-derived liver metastases compared to control liver metastases (Fig. 6d, *P* < 0.0001). Furthermore, compared with those in control liver metastases, IF revealed reduced Glut1 protein levels in CMTM6-knockdown liver metastases (Fig. 6e, *P* < 0.0001). Moreover, WB confirmed that the average Glut1 level was lower but the average activated-caspase 3 level was higher in CMTM6-knockdown liver metastases than in control metastases (Fig. 6f, *P* < 0.05). Thus, CMTM6 is required for maintaining Glut1 protein levels in CRC and CRC liver metastases in mice.

### CMTM6 knockdown blocks the secretion of cytokines/chemokines by MC38 cells

**Fig. 7:**
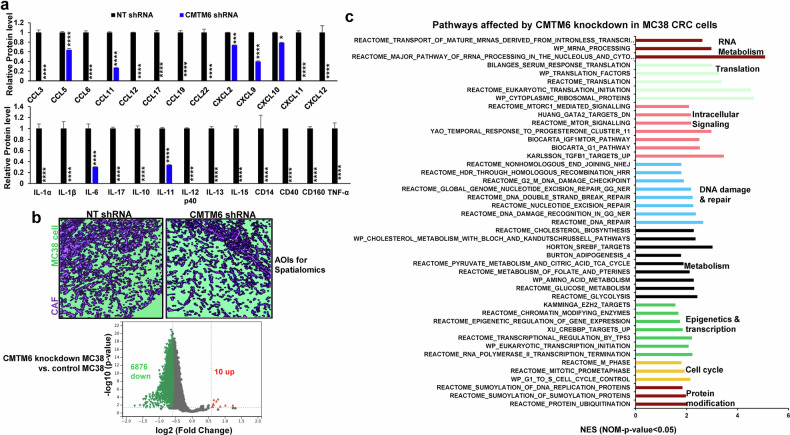
CMTM6 knockdown blocks the secretion of cytokines/chemokines by CRC cells and induces global transcriptomic changes in CRC liver metastases. **a** The conditioned media of control and CMTM6-knockdown MC38 cells were analyzed with a Proteome Profiler Mouse XL Cytokine Array Kit (R&D Systems). CMTM6 knockdown reduced the secretion of cytokines/chemokines by MC38 cells. *, *P* < 0.05; ***, *P* < 0.001, ****P < 0.0001 by *t* test, *n* = 4. **b** Upper panel, sec*t*ions of MC38 cell-derived liver metastases were subjected to spatial transcriptomics with NanoString GeoMx DSP. Two representative areas of interest (AOIs) that were selected for study are shown. MC38 cells were labeled with an anti-pan cytokeratin antibody (green by the software), and cancer-associated fibroblasts (CAFs) were labeled with an anti-desmin antibody (purple). Lower panel, a volcano plot showing 6876 downregulated and 10 upregulated transcripts in MC38 cells as the result of targeting CMTM6 of MC38 cells. The horizontal line represents *P* < 0.05, and the vertical lines represent a fold change of 1.5. **c** The pathways in MC38 cells that were affe**c**ted by CMTM6 knockdown were identified by GSEA with the M2 pathways based on NES > 1 and *P* < 0.05. NES normalized enrichment score.

To explore additional mechanisms by which CMTM6 promotes CRC liver metastasis, we focused on MC38 cell-derived cytokines and chemokines because they not only promote cancer proliferation and growth but also establish a prometastatic microenvironment by recruiting or activating other cells in a paracrine fashion. Conditioned medium was collected from control or CMTM6-knockdown MC38 cells for cytokine/chemokine profiling using a Proteome Profiler Mouse XL Cytokine Array Kit (R&D Systems). In total, 73 out of 111 cytokine/chemokine targets were detected, and 60 were significantly downregulated by CMTM6 knockdown (Supplementary Fig. 3, *P* < 0.05). The CMTM6 targets can be divided into different groups: (1) mitogens such as PDGF-BB, Amphiregulin, and TGFα; (2) cytokines/chemokines that are related to inflammation, such as IL-1α, IL-1β, IL6, IL11, CD14, CD160, Chemerin, Chitinase-3-like protein 1 (CHI3L1), Flt-3 ligands, G-CSF, M-GSF, ICAM-1, CD62E, CD62P, and VCAM1; (3) factors that are involved in the immunosuppressive tumor microenvironment, such as IL6, IL10, TNFα, CCL17, and IL15; and (4) factors that control endothelial cell survival and angiogenesis, such as CXCL12, angiopoinetin-1, GAS6, and MMP2 (Supplementary Fig. 3). Some targets are shown in Fig. 7a. Thus, by controlling the secretion of cytokines/chemokines by CRC cells, CMTM6 in CRC cells can remodel the hepatic tumor microenvironment to promote CRC liver metastasis.

### CMTM6 promotes the development of an immunosuppressive microenvironment for CRC liver metastasis

Cancer-associated fibroblasts (CAFs) and various immune cells in the liver are critical determinants of CRC liver metastasis. We previously reported that the CAFs of MC38 cell-derived liver metastases exhibit active PDGF signaling and interleukin signaling pathways, which are important for CAF activation^15^. Since CMTM6 knockdown blocked the secretion of PDGF-BB and numerous interleukins by MC38 cells (Supplementary Fig. 3, *P* < 0.0001), we quantified the CAF density of MC38 cell-derived liver metastases. IF staining for α-smooth muscle actin (αSMA), which is a marker of CAFs, revealed that the CAF density was significantly lower in CMTM6-knockdown liver metastases than in control liver metastases (Supplementary Fig. 4, *P* < 0.05). CAFs facilitate cancer immune evasion by forming a physical barrier to prevent immune cells from entering tumors and by producing immune regulatory molecules, such as TGFβ1 and PD-L1, to suppress the antitumor activity of immune cells^31,33,44,45^. Thus, we profiled the immune components of MC38 cell-derived liver metastases by performing IF, and the results revealed that the densities of CD8a+ T cells, NK1.1 + NK/NKT cells, Granzyme B+ cytotoxic lymphoid cells, and CD20 + B cells were increased in CMTM6-knockdown liver metastases compared to control liver metastases (Supplementary Figs. 4, 5, *P* < 0.001). In contrast, the numbers of FoxP3+ regulatory T cells and F4/80+ macrophages were decreased in CMTM6-knockdown liver metastases (Supplementary Fig. 5, *P* < 0.0001). Thus, CMTM6 in CRC cells promotes an immunosuppressive microenvironment by recruiting CAFs into liver metastases and excluding cytotoxic lymphocytes from CRC liver metastases.

### Targeting CMTM6 of MC38 cells alters the transcriptome of MC38 cells within the liver metastases, as revealed by spatial transcriptomics

To determine whether and how CMTM6 knockdown influenced the transcriptome of MC38 cell-derived liver metastases, we performed spatial transcriptomics using the NanoString GeoMx Digital Spatial Profiler (GeoMx DSP) and GeoMx Mouse Whole Transcriptome Atlas Panel that allows the detection of 20,177 murine transcripts^15^. Control MC38 cell-derived liver metastases from 3 different mice and CMTM6-knockdown metastases from 3 mice were selected for the study; anti-pan cytokeratin and anti-desmin antibodies were used to label MC38 cells and CAFs by IF staining (Fig. 7b upper panel). In total, 14 areas of interest (AOIs) in control metastases and 13 AOIs in CMTM6-knockdown metastases were selected for data acquisition and analysis, which led to the detection of 19,616 transcripts in MC38 cells from each AOI. Compared to the transcriptome of HCT116 cells (Fig. 2a), the spatialomic data, as shown in a volcano plot, demonstrated a remarkable shift toward the left (Fig. 7b lower panel), suggesting a transcriptome-wide downregulation of gene expression in CMTM6-knockdown MC38 cell-derived liver metastases compared to that in control liver metastases. When a fold change of 1.5 was set as the threshold, 6876 transcripts were found to be downregulated and 10 were found to be upregulated in CMTM6-knockdown MC38 cells in the liver metastases (*P* < 0.05). GSEA analysis with the mouse molecular signature database (M2 gene sets) revealed that transcriptomic changes impacted the biological processes of MC38 cells, including RNA metabolism, translation, intracellular signaling, DNA damage and repair, metabolism, epigenetics and gene transcription, the cell cycle, and protein modification (Fig. 7c). For example, 175 transcripts in the Reactome G2-G2/M phase and 61 transcripts in the Reactome glycolysis were affected by CMTM6 knockdown in MC38 cells (Supplementary Fig. 6). Since the secretion of 60 cytokines/chemokines was blocked by CMTM6 knockdown (Supplementary Figure 3), we compared the transcript levels of these cytokines/chemokines. Among the 59 transcripts that were analyzed, 50 were significantly downregulated in CMTM6-knockdown MC38 cell-derived liver metastases compared to control MC38 cell-derived liver metastases (Supplementary Fig. 7a, *P* < 0.05), whereas 9 transcripts were not downregulated (Supplementary Fig. 7b, *P* > 0.05). Thus, while the majority of the cytokine/chemokine targets of CMTM6 are regulated by CMTM6 at the transcriptional level, a small portion of these targets is regulated at the posttranscriptional level in CRC cells.

### Targeting CMTM6 in MC38 cells alters the transcriptome of CAFs, as revealed by spatial transcriptomics

**Fig. 8:**
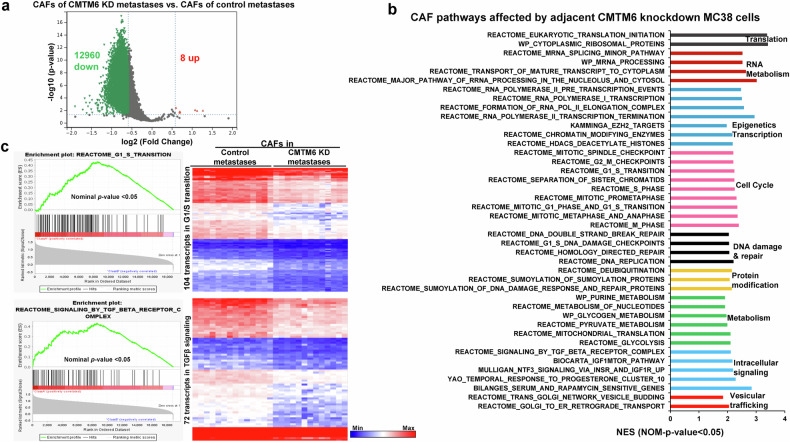
Targeting CMTM6 in MC38 cells alters the transcriptome of adjacent CAFs. **a** Volcano plot showing 12960 downregulated and 8 upregulated transcripts in CAFs of CMTM6-knockdown liver metastases compared to control liver metastases. The horizontal line represents *P* < 0.05, and the vertical lines represent a fold change of 1.5**. b** The CAF pathways and cellular processes that were affected by targeting CMTM6 in MC38 cells were identified by GSEA (NES > 1 and *P* < 0.05, *n* = 14,13). **c** Two CAF gene sets whose trans**c**ripts were affected by the targeting of CMTM6 in MC38 cells are shown. Transcript enrichment is shown by an enrichment plot (left panel), and gene expression levels are shown by a heatmap (right panel). *n* = 14,13. The bar represents the minimum (blue) to the maximum expression level (red).

A spatialomics study also detected 18,856 transcripts in CAFs from each AOI. We compared the transcriptome of control CAFs to that of CAFs from CMTM6-knockdown MC38 cell-derived liver metastases, and the results are shown in a volcano plot (Fig. 8a). The data also showed a remarkable shift toward the left, suggesting a transcriptome-wide downregulation of gene expression in the CAFs of CMTM6-knockdown liver metastases (Fig. 8a). When a fold change of 1.5 was set as the threshold, 12,960 transcripts were found to be downregulated and 8 were found to be upregulated in the CAFs of CMTM6-knockdown metastases (Fig. 8a*P* < 0.05). GSEA revealed that these transcriptomic changes affected biological processes in CAFs, such as translation, RNA metabolism, epigenetics and gene transcription, the cell cycle, DNA damage and repair, protein modification, metabolism, intracellular signaling, and vesicular trafficking (Fig. 8b). For example, the expression of 104 transcripts in the Reactome G1/S transition (Fig. 8c upper panel) and 92 transcripts in the Reactome metabolism of nucleotides in CAFs were affected (Supplementary Fig. 8). TGFβ signaling is critical for CAF activation^36,46^. Seventy-two transcripts related to Reactome signaling by the TGFbeta receptor complex were suppressed (Fig. 8c, lower panel), which was consistent with the results of IF staining for αSMA showing that the CAF density was lower in CMTM6-knockdown liver metastases than in control liver metastases (Supplementary Fig. 4, *P* < 0.01). Thus, CMTM6 in CRC cells regulates the transcriptome of both CRC cells and adjacent CAFs to promote colorectal liver metastasis.

## Discussion

The majority of previous studies on the role of CMTM6 have focused on how CMTM6 maintains PD-L1 protein levels and targets PD-L1 to the PM, which are processes that are closely related to immune evasion in cancer. This study focused on the role of CMTM6 in glycolysis and the transcriptome, and this study revealed that CMTM6 is required for maintaining the transcriptome of CRC cells, targeting Glut1 to the PM for glycolysis, and promoting cell cycle progression in CRC cells. In vivo, CMTM6 is required for subcutaneous CRC growth in immunocompromised mice and for MC38 cell-derived CRC liver metastatic growth in C57BL/6 mice. By performing targeted proteomics and IF studies, we further showed that CMTM6 in CRC cells controls the secretion of 60 cytokines/chemokines and recruits CAFs to establish an immunosuppressive microenvironment for CRC liver metastases. Moreover, spatial transcriptomics confirmed that global transcriptomic changes occurred in CAFs of CRC liver metastases as a result of targeting CMTM6 in MC38 cells. Thus, CMTM6 plays multifaceted roles in CRC liver metastasis.

GSEA revealed that two pathways, the Fischer G2/M cell cycle pathway and the Reactome glucose metabolism pathway, were affected by CMTM6 knockdown in HCT116 cells. Cell cycle analysis confirmed that CMTM6 knockdown disrupted cell cycle progression, as the number of CMTM6-knockdown CRC cells in the G2 phase of the cell cycle was increased. Our findings suggest the requirement of CMTM6 for CRC cell cycle progression, which is different from the role of CMTM6 in hepatocellular carcinoma (HCC)^25^. Wang et al. showed that CMTM6 suppresses the proliferation of HCC cells by blocking the G1-to-S phase transition by stabilizing p21 and inactivating the pRB/E2F pathway^25^. Interestingly, patient data from the TCGA support the conclusion that CMTM6 plays different roles in CRC and HCC; CMTM6 expression is upregulated in patient CRC tissues but downregulated in HCC tissues compared to normal control tissues. Thus, the role and mechanism of CMTM6 appear to be determined by the specific type of cancer.

Targeted proteomics was used to identify 60 cytokines/chemokines as CMTM6 targets because their secretion was blocked by CMTM6 knockdown. Members of the Rab small GTPase family regulate membrane trafficking, including vesicle formation, vesicle movement inside the cell, and membrane fusion, and thus, they regulate the transport of cargo proteins from the Golgi to the PM and their secretion out of the cell; this process is termed exocytosis^47^. Although the role of Rab11 in exocytosis has been demonstrated^42,48^, other Rabs, such as Rab3, Rab4, and Rab6, are required for exocytosis^49,50^. Since some of the cytokine/chemokine targets of CMTM6 are posttranscriptionally regulated by CMTM6 (Supplementary Fig. 7b), we hypothesize that in addition to the function of Rab11, CMTM6 may be important for the function of other members of the Rab small GTPase family that are involved in protein trafficking and exocytosis.

Spatial transcriptomics revealed that global transcriptomic changes occurred in CMTM6-knockdown MC38 cell-derived liver metastases compared to control metastases, and these changes may be explained by the following reasons. (1) Some glycolysis-derived metabolites and intermediaries directly participate in DNA and RNA synthesis or regulate gene transcription via epigenetic mechanisms. For example, the pentose phosphate pathway, which is a branch of the glycolysis pathway, generates ribose 5-phosphate (R5P), which is used in the synthesis of nucleotides and nucleic acids. Serine, which is synthesized de novo from the glycolysis intermediate 3-phosphoglyceric acid, enters folate metabolism (one-carbon metabolism) to form 5,10-methylene tetrahydrofolate, which is required for DNA, RNA, and methionine synthesis. *S*-Adenosyl methionine is a methyl donor that is involved in the methylation of DNA, RNA, and histones. Moreover, acetyl-CoA, which is another glycolytic metabolite, is an acetyl donor that is involved in the histone acetylation that is required for gene transcription. Targeting CMTM6 led to the suppression of glycolysis and the production of these metabolites, thereby influencing the transcription of genes in CRC cells. (2) PM receptors are required for signal transduction and gene transcription in the nucleus. Many PM receptors are targeted to the PM via a Rab11-dependent mechanism. CMTM6 knockdown suppressed Rab11 mRNA levels and Rab11 activity, so PM receptor-mediated signal transduction and transcription programs may be affected in CMTM6-knockdown cells. (3) CMTM6-knockdown liver metastases cannot efficiently recruit CAFs. CAFs are another major producer of growth factors, cytokines, and chemokines that in turn act on CRC cells to modulate CRC gene transcription via paracrine mechanisms. Thus, the lack of CAFs and CAF-derived factors in the hepatic tumor microenvironment could impact the transcriptome of CRC cells in liver metastases.

In summary, we identified a mechanism underlying the Warburg effect by which CMTM6 forms a complex with Glut1 and Rab11 in endosomes, and this complex is required for the Rab11-dependent transport of Glut1 to the PM, glucose uptake, and glycolysis in CRC cells. Functionally, CMTM6 is required for maintaining the transcriptome, cell cycle progression, and liver metastasis of CRC in mice. Since the Warburg effect is a common metabolic feature of cancer cells, understanding the structural basis of the CMTM6/Glut1 interaction may provide opportunities for the design of drugs that target the CMTM6/Glut1 interaction. In this regard, future studies could focus on using structural biology approaches to determine how CMTM6 binds to Glut1 and in order to disrupt the structure of the interface that mediates the CMTM6/Glut1 interaction. The information obtained will help us identify appropriate strategies, such as screening small molecule libraries or drug libraries, to search for compounds that can be used to disrupt the Warburg effect and suppress CRC liver metastasis.

## Supplementary information

Supplementary Information

## Data Availability

RNA sequencing data: GSE252571: token: whelyowwdnsvnuj. Spatialomics data: GSE253937: token: chkfwquadlgtnwl; GSE253938: token: ujozymyqhxkvpmj. The datasets that were generated and/or analyzed during this study are available from the corresponding author upon reasonable request.

## References

[1] Tsilimigras, D. I. et al. Liver metastases. *Nat. Rev. Dis. Prim.* **7**, 27 (2021). 33859205 10.1038/s41572-021-00261-6

[2] Faubert, B., Solmonson, A. & DeBerardinis, R. J. Metabolic reprogramming and cancer progression. *Science* **368**, eaaw5473 (2020). 32273439 10.1126/science.aaw5473 PMC7227780

[3] Yang, F. et al. Metabolic reprogramming and its clinical implication for liver cancer. *Hepatology* **78**, 1602–1624 (2023). 36626639 10.1097/HEP.0000000000000005 PMC10315435

[4] Vander Heiden, M. G., Cantley, L. C. & Thompson, C. B. Understanding the Warburg effect: the metabolic requirements of cell proliferation. *Science* **324**, 1029–1033 (2009). 19460998 10.1126/science.1160809 PMC2849637

[5] Potter, M., Newport, E. & Morten, K. J. The Warburg effect: 80 years on. *Biochem Soc. Trans.* **44**, 1499–1505 (2016). 27911732 10.1042/BST20160094 PMC5095922

[6] Izuishi, K. et al. Molecular mechanisms of [18F]fluorodeoxyglucose accumulation in liver cancer. *Oncol. Rep.* **31**, 701–706 (2014). 24297035 10.3892/or.2013.2886

[7] Akiyoshi, T. et al. Comparison of preoperative whole-body positron emission tomography with MDCT in patients with primary colorectal cancer. *Colorectal Dis.* **11**, 464–469 (2009). 18637927 10.1111/j.1463-1318.2008.01643.x

[8] Szablewski, L. Expression of glucose transporters in cancers. *Biochim. Biophys. Acta* **1835**, 164–169 (2013). 23266512 10.1016/j.bbcan.2012.12.004

[9] Haber, R. S. et al. GLUT1 glucose transporter expression in colorectal carcinoma: a marker for poor prognosis. *Cancer* **83**, 34–40 (1998). 9655290 10.1002/(sici)1097-0142(19980701)83:1<34::aid-cncr5>3.0.co;2-e

[10] Cooper, R. et al. Glucose transporter-1 (GLUT-1): a potential marker of prognosis in rectal carcinoma? *Br. J. Cancer* **89**, 870–876 (2003). 12942120 10.1038/sj.bjc.6601202 PMC2394489

[11] Wieman, H. L., Wofford, J. A. & Rathmell, J. C. Cytokine stimulation promotes glucose uptake via phosphatidylinositol-3 kinase/Akt regulation of Glut1 activity and trafficking. *Mol. Biol. Cell* **18**, 1437–1446 (2007). 17301289 10.1091/mbc.E06-07-0593 PMC1838986

[12] Cohen, M. et al. Live imaging of GLUT2 glucose-dependent trafficking and its inhibition in polarized epithelial cysts. *Open Biol.* **4**, 140091 (2014). 25056286 10.1098/rsob.140091 PMC4118605

[13] Uhlig, M., Passlack, W. & Eckel, J. Functional role of Rab11 in GLUT4 trafficking in cardiomyocytes. *Mol. Cell Endocrinol.* **235**, 1–9 (2005). 15866422 10.1016/j.mce.2005.02.004

[14] Du, W. et al. IFN-gamma/mTORC1 decreased Rab11 in Schwann cells of diabetic peripheral neuropathy, inhibiting cell proliferation via GLUT1 downregulation. *J. Cell Physiol.* **235**, 5764–5776 (2020). 31970777 10.1002/jcp.29510

[15] Wang, Y. et al. Targeting Src SH3 domain-mediated glycolysis of HSC suppresses transcriptome, myofibroblastic activation, and colorectal liver metastasis. *Hepatology*10.1097/HEP.0000000000000763 (2024). 10.1097/HEP.0000000000000763 PMC11266532 38271673

[16] Wu, J., Li, L., Wu, S. & Xu, B. CMTM family proteins 1-8: roles in cancer biological processes and potential clinical value. *Cancer Biol. Med*. **17**, 528–542 (2020). 32944388 10.20892/j.issn.2095-3941.2020.0032 PMC7476098

[17] Burr, M. L. et al. CMTM6 maintains the expression of PD-L1 and regulates anti-tumour immunity. *Nature* **549**, 101–105 (2017). 28813417 10.1038/nature23643 PMC5706633

[18] Mezzadra, R. et al. Identification of CMTM6 and CMTM4 as PD-L1 protein regulators. *Nature* **549**, 106–110 (2017). 28813410 10.1038/nature23669 PMC6333292

[19] Gao, H. et al. CMTM6 as a potential therapy target is associated with immunological tumor microenvironment and can promote migration and invasion in pancreatic adenocarcinoma. *Funct. Integr. Genomics* **23**, 306 (2023). 37726578 10.1007/s10142-023-01235-5 PMC10509136

[20] Wei, L., Wei, Q., Yang, X. & Zhou, P. CMTM6 knockdown prevents glioma progression by inactivating the mTOR pathway. *Ann. Transl. Med.* **10**, 181 (2022). 35280358 10.21037/atm-21-6894 PMC8908166

[21] Chen, L. et al. Targeting CMTM6 Suppresses Stem Cell-like Properties And Enhances Antitumor Immunity In Head And Neck Squamous Cell Carcinoma. *Cancer Immunol. Res* **8**, 179–191 (2020). 31771985 10.1158/2326-6066.CIR-19-0394

[22] Huang, X. et al. CMTM6 as a candidate risk gene for cervical cancer: Comprehensive bioinformatics study. *Front Mol. Biosci.* **9**, 983410 (2022). 36589225 10.3389/fmolb.2022.983410 PMC9798917

[23] Liu, Q. et al. CMTM6 promotes hepatocellular carcinoma progression through stabilizing beta-catenin. *Cancer Lett.* **583**, 216585 (2023). 38101607 10.1016/j.canlet.2023.216585

[24] Huang, X. et al. CMTM6 promotes migration, invasion, and EMT by interacting with and stabilizing vimentin in hepatocellular carcinoma cells. *J. Transl. Med* **19**, 120 (2021). 33757532 10.1186/s12967-021-02787-5 PMC7989033

[25] Huang, Y. et al. CMTM6 inhibits tumor growth and reverses chemoresistance by preventing ubiquitination of p21 in hepatocellular carcinoma. *Cell Death Dis.* **13**, 251 (2022). 35304440 10.1038/s41419-022-04676-1 PMC8933468

[26] Liu, C. et al. IQGAP1 suppresses TbetaRII-mediated myofibroblastic activation and metastatic growth in liver. *J. Clin. Invest* **123**, 1138–1156 (2013). 23454766 10.1172/JCI63836 PMC3582119

[27] Dou, C. et al. P300 acetyltransferase mediates stiffness-induced activation of hepatic stellate cells into tumor-promoting myofibroblasts. *Gastroenterology* **154**, 2209–2221.e2214 (2018). 29454793 10.1053/j.gastro.2018.02.015 PMC6039101

[28] Wang, Y. et al. p300 acetyltransferase is a cytoplasm-to-nucleus shuttle for SMAD2/3 and TAZ nuclear transport in transforming growth factor beta-stimulated hepatic stellate cells. *Hepatology* **70**, 1409–1423 (2019). 31004519 10.1002/hep.30668 PMC6783326

[29] Kang, N. et al. Focal adhesion assembly in myofibroblasts fosters a microenvironment that promotes tumor growth. *Am. J. Pathol.* **177**, 1888–1900 (2010). 20802179 10.2353/ajpath.2010.100187 PMC2947284

[30] Decker, N. K. et al. Nitric oxide regulates tumor cell cross-talk with stromal cells in the tumor microenvironment of the liver. *Am. J. Pathol.* **173**, 1002–1012 (2008). 18755846 10.2353/ajpath.2008.080158 PMC2543069

[31] Chen, Y. et al. Focal adhesion kinase promotes hepatic stellate cell activation by regulating plasma membrane localization of TGFbeta receptor 2. *Hepatol. Commun.* **4**, 268–283 (2020). 32025610 10.1002/hep4.1452 PMC6996408

[32] Liu, D. et al. Protein diaphanous homolog 1 (Diaph1) promotes myofibroblastic activation of hepatic stellate cells by regulating Rab5a activity and TGFbeta receptor endocytosis. *FASEB J.* **34**, 7345–7359 (2020). 32304339 10.1096/fj.201903033R PMC7686927

[33] Sun, L. et al. PD-L1 promotes myofibroblastic activation of hepatic stellate cells by distinct mechanisms selective for TGF-beta receptor I versus II. *Cell Rep.* **38**, 110349 (2022). 35139382 10.1016/j.celrep.2022.110349 PMC8903892

[34] Tu, K. et al. Vasodilator-stimulated phosphoprotein promotes activation of hepatic stellate cells by regulating Rab11-dependent plasma membrane targeting of transforming growth factor beta receptors. *Hepatology* **61**, 361–374 (2015). 24917558 10.1002/hep.27251 PMC4262723

[35] Liu, C. et al. PDGF receptor-alpha promotes TGF-beta signaling in hepatic stellate cells via transcriptional and posttranscriptional regulation of TGF-beta receptors. *Am. J. Physiol. Gastrointest. Liver Physiol.* **307**, G749–G759 (2014). 25169976 10.1152/ajpgi.00138.2014 PMC4187064

[36] Kang, N., Shah, V. H. & Urrutia, R. Membrane-to-nucleus signals and epigenetic mechanisms for myofibroblastic activation and desmoplastic stroma: Potential therapeutic targets for liver metastasis? *Mol. cancer Res.* **13**, 604–612 (2015). 25548101 10.1158/1541-7786.MCR-14-0542 PMC4398610

[37] Affo, S. et al. Promotion of cholangiocarcinoma growth by diverse cancer-associated fibroblast subpopulations. *Cancer Cell* **39**, 866–882.e811 (2021). 33930309 10.1016/j.ccell.2021.03.012 PMC8241235

[38] Bhattacharjee, S. et al. Tumor restriction by type I collagen opposes tumor-promoting effects of cancer-associated fibroblasts. *J. Clin. Invest* **131**, e146987 (2021). 33905375 10.1172/JCI146987 PMC8159701

[39] Wan, P. et al. Guidance receptor promotes the asymmetric distribution of exocyst and recycling endosome during collective cell migration. *Development* **140**, 4797–4806 (2013). 24198275 10.1242/dev.094979

[40] Butterworth, M. B. et al. Rab11b regulates the trafficking and recycling of the epithelial sodium channel (ENaC). *Am. J. Physiol. Ren. Physiol.* **302**, F581–F590 (2012). 10.1152/ajprenal.00304.2011 PMC3353647 22129970

[41] Grant, B. D. & Donaldson, J. G. Pathways and mechanisms of endocytic recycling. *Nat. Rev. Mol. Cell Biol.* **10**, 597–608 (2009). 19696797 10.1038/nrm2755 PMC3038567

[42] Takahashi, S. et al. Rab11 regulates exocytosis of recycling vesicles at the plasma membrane. *J. Cell Sci.* **125**, 4049–4057 (2012). 22685325 10.1242/jcs.102913

[43] Campa, C. C. et al. Rab11 activity and PtdIns(3)P turnover removes recycling cargo from endosomes. *Nat. Chem. Biol.* **14**, 801–810 (2018). 29915378 10.1038/s41589-018-0086-4

[44] Chakravarthy, A., Khan, L., Bensler, N. P., Bose, P. & De Carvalho, D. D. TGF-beta-associated extracellular matrix genes link cancer-associated fibroblasts to immune evasion and immunotherapy failure. *Nat. Commun.* **9**, 4692 (2018). 30410077 10.1038/s41467-018-06654-8 PMC6224529

[45] Jenkins, L. et al. Cancer-associated fibroblasts suppress CD8+ T-cell infiltration and confer resistance to immune-checkpoint blockade. *Cancer Res.* **82**, 2904–2917 (2022). 35749591 10.1158/0008-5472.CAN-21-4141 PMC9379365

[46] Kang, N., Gores, G. J. & Shah, V. H. Hepatic stellate cells: partners in crime for liver metastases? *Hepatology* **54**, 707–713 (2011). 21520207 10.1002/hep.24384 PMC3145026

[47] Fischer von Mollard, G., Stahl, B., Li, C., Sudhof, T. C. & Jahn, R. Rab proteins in regulated exocytosis. *Trends Biochem Sci.* **19**, 164–168 (1994). 8016866 10.1016/0968-0004(94)90278-x

[48] Escrevente, C., Bento-Lopes, L., Ramalho, J. S. & Barral, D. C. Rab11 is required for lysosome exocytosis through the interaction with Rab3a, Sec15 and GRAB. *J. Cell Sci.* **134**, jcs246694 (2021). 34100549 10.1242/jcs.246694 PMC8214760

[49] Darchen, F. & Goud, B. Multiple aspects of Rab protein action in the secretory pathway: Focus on Rab3 and Rab6. *Biochimie* **82**, 375–384 (2000). 10865125 10.1016/s0300-9084(00)00219-4

[50] Ohnishi, H. et al. Involvement of Rab4 in regulated exocytosis of rat pancreatic acini. *Gastroenterology* **116**, 943–952 (1999). 10092317 10.1016/s0016-5085(99)70078-8

