# Supplementary material for: CMTM6 mediates the Warburg effect and promotes the liver metastasis of colorectal cancer
Source: Exp Mol Med. 2024 Sep 2;56(9):2002–15. doi: 10.1038/s12276-024-01303-1 (PMC11447025; doi:10.1038/s12276-024-01303-1)
Supplement: Supplementary file 1 — Supplementary Information [file 12276_2024_1303_MOESM1_ESM.pdf]

Supplementary Materials and Methods

Cell lines. Human colorectal cancer (CRC) cell lines, HCT116 and KM12L4, were purchased from ATCC (Manassas, VA, USA) and they were authenticated by short-tandem repeat DNA profiling by Genetica DNA Laboratories (Cincinnati, OH). MC38 mouse colorectal cancer cells were kindly provided by Dr. Steven A. Rosenberg at the National Cancer Institute[1-3]. Cells were monitored for mycoplasma infection and they were free of infection during the experiments.

Antibodies and reagents. Antibodies for Western blot (WB): anti-Glut1 (1:20,000; ab115730 Abcam, Cambridge, UK), anti-CMTM6 antibody (1:1000; 903295 Cell Signaling Technology, Danvers, MA), anti-Rab11 (1:500; 5589 Cell Signaling Technology), anti-active Rab11 antibody (1:500; 26919 NewEast Bioscience, Kelayres, PA), anti-cleaved caspase 3 (D175) (1:1000; 9664 Cell Signaling Technology), anti-GAPDH (1:1000; 2118 Cell Signaling Technology), anti-DYKDDDDK (1:1000; 2368 Cell Signaling Technology); and anti-HA (C29F4) (1:1000; 3724 Cell Signaling Technology).

Antibodies for immunofluorescence (IF): anti-CMTM6 (1:500; 90329 Cell Signaling Technology), anti-Glut1(1:1000; ab238050 Abcam), anti-Glut1(1:4000; ab115730 Abcam), anti-HA 647-conjugated (1:200; sc-7392 Santa Cruz Biotechnology, Dallas, TX); Anti-FLAG (M2) (1:4000; F1804 MilliporeSigma, Burlington, MA), anti-cleaved caspase 3 (D175) (1:1000: 9664 Cell Signaling Technology), anti-Ki67 (ab15580 abcam), anti-Desmin (Y66) (ab32362 Abcam), and anti-pan cytokeratin (C11) (1:500; sc-8018 Santa Cruz Biotechnology), anti-CD8a (1:150; ab209775 Abcam), anti-CD20 (1: 150; ab64088 Abcam), anti-Granzyme B (1:250; AF1865 R & D Systems, Minneapolis, MN), anti-F4/80 (1:200; sc-25830 Santa Cruz Biotechnology), anti-NK-

1.1 (1:150; 553165 BD Pharmingen, Becton, Dickinson, and Company, NJ), anti-FoxP3 (1:100; sc-166212 Santa Cruz Biotechnology).

Bafilomycin (1334 Tocris, Minneapolis MN), MG132 (10012628 Cayman Chemical Company, Ann Arbor, MI), E64d (4545 Tocris), and Pepstatin A (51648 Calbiochem, Millipore Sigma). If a mouse monoclonal antibody was used to stain mouse liver metastases, the M.O.M. Immunodetection Kit, Fluorescein, was used (FMK-2201 Vector Laboratories, Newark, CA).

Viral constructs and viral transduction of CRC cells. Lentiviral constructs encoding CMTM6 shRNA were obtained from the Sigma MISSION shRNA library (MilliporeSigma). CMTM6 shRNA1 (TRCN0000129143) and CMTM6 shRNA2 (TRCN0000130201) target human CMTM6; Cmtm6 shRNA1 (TRCN0000121354), Cmtm6 shRNA2 (TRCN0000121355), and Cmtm6 shRNA3 (TRCN0000121356) target mouse CMTM6. A construct encoding a non-targeting shRNA was used as a control (SHC202; MilliporeSigma). CMTM6 cDNA (OHu24864 GenScript Biotech Piscataway, NJ) was inserted into the retroviral pMMP vector by standard PCR-based subcloning techniques with HA added to its C terminus. The pMMPRab11-FLAG vector was created in the lab previously[4], and based on the pMMPRab11-FLAG vector, pMMPRab11Q70L-FLAG was generated by Q5 Site-Directed Mutagenesis (#E0554 New England Biolabs Ipswich, MA). All constructs were confirmed by sequencing and WB before the experiments.

Replication-defective lentiviruses and retroviruses were generated by transfecting 293T/17 cells with three specific plasmids by the Effectene Transfection Reagent (301425 Qiagen, Tegelen, Netherlands), as previously described[1, 5, 6]. Cell culture supernatants containing virial particles were collected 48 and 72 hours later and the viruses were stored at -80°C. Viral transduction of the cells was done by incubating the cells at 37°C overnight with virus-containing supernatant (1:1

dilution by completed DMEM medium) supplemented with polybrene (8 μg/mL). Cells were collected 72 hours later for subsequent studies.

Cell proliferation assay and 3D tumor spheroid culture. CRC cells were seeded into a 96-well plate at a density of 5×10^3 per well and measurements were made at 5 time points, 0, 24, 48, 72 and 96 hours, with CellTiter 96® AQ_{ueous} One Solution Cell Proliferation Assay kit according to the manufacturer's instructions (G3582 Promega, Madison, WI). Briefly, 20 µL of MTS reagent was added into each well containing 100 µL medium and the absorbance at 490 nm was measured by an Epoch microplate reader (11-120-570 Thermo Fisher Scientific, Waltham, MA) after the cells were incubated at 37°C for 3 hours. Each group had 5 replicates.

The Nunclon Sphera 96-well U-bottom ultra-low attachment cell culture plates were used to induce 3D tumoroids (174925 Thermo Fisher Scientific). HCT116 cells were seeded at a density of 3×10^3 cells per well, and MC38 cells were seeded at 5×10^3 cells per well, and the cells self-assembled into one 3D spheroid in each well 48 hours later. More than 10 spheroids per group were photographed under an EVOS FL imaging system (12-563-631 Thermo Fisher Scientific), and they were calculated by the equation: volume = $(\text{width})^2 \times \text{length} / 2$.

Cell viability in the tumoroids was analyzed using LIVE/DEAD™ Viability/Cytotoxicity Kit (L3224 Invitrogen, Thermo Fisher Scientific). In brief, cell-permeant Calcein AM was first mixed with Ethidium homodimer-1 dye, and 20 μL of the dye mix were added into each well of the culture plate. Calcein AM was converted to intensely green fluorescent calcein (ex/em ~495 nm/~515 nm) in live cells, and Ethidium homodimer-1 produced a bright red fluorescence (ex/em ~495 nm/~635 nm) after binding to nucleic acids of dead cells. After incubation with the dye mix for 15 minutes at 20°C - 25°C, green and red fluorescent signals in the tumoroids were captured

under an Axio observer microscope with a 20× lens (Zeiss, Oberkochen, Germany) and the Zen Blue software (Zeiss, Germany).

The compactness of tumor spheroids was evaluated by incubating the tumoroids with Dil-Dye (D3911 ThermoFisher Scientific). Dil-Dye was added into each well of the plate (2.5 µg/mL), and the tumoroids were imaged 4 hours later under the Axio observer with the Zen Blue software.

Cell membrane integrity assessment by Propidium iodide (PI) staining. Control and CMTM6 knockdown HCT116 cells in cell culture dishes were incubated with PBS containing 1 µg/mL PI. Cells positive for PI staining (red fluorescent) were immediately examined and counted under a fluorescence microscope with a 20x lens, and the percentage of PI positive cells in each group was calculated.

Colony formation assay. Colony formation assay was used to assess the ability of a single cell to form a colony in culture. HCT116 cells transduced with NT shRNA lentiviruses or CMTM6 shRNA lentiviruses were seeded into the well of a 6-well-culture plate (500 cells per well). HCT116 colonies were induced by incubation of the cells in DMEM culture medium containing 10% fetal bovine serum in a standard cell culture incubator supplied with 5% CO_2. Two weeks later, the colonies were fixed in 4% paraformaldehyde and stained by crystal violet (0.5% w/v) for 2 hours. The number of the colonies in each well was manually counted, and the size of the colonies was quantitated with the ImageJ software (NIH).

Cell cycle analysis. Control and CMTM6 knockdown CRC cells were detached from a cell culture plate by trypsinization. After the cells were washed once with PBS, 70% ethanol was added to fix the cells for 20 minutes. Cells were next incubated with RNase A (100 µg/mL diluted in PBS) for 15 minutes at room temperature to remove RNA. Lastly, the cells were incubated with PI (50

μg/mL in PBS) for 30 minutes in dark. Flow cytometry was performed with a BD LSRFortessaTM X-20 Cell Analyzer, and the data were analyzed by the FlowJo_v10.8.1 software.

β-galactosidase staining. Senescent CRC cells were detected by a Senescence β-Galactosidase Staining Kit (9860 Cell Signaling Technology). In brief, HCT116 cells transduced with control or CMTM6 shRNA lentiviruses were harvested and fixed in the fixative solution for 15 minutes at room temperature. The cells were next stained with β-galactosidase staining solution overnight at 37ºC. Cells stained blue were captured under a Leica inverted microscope and the percentage of blue cells was calculated in randomly selected microscopic fields[2].

Western Blot analysis (WB). Proteins were extracted from the cells or tumors by using the RIPA lysis buffer containing 1% Nonidet P-40, 1% sodium deoxycholate, and 0.1% SDS, supplemented with a protease inhibitor cocktail (A32965, Thermo Fisher Scientific). After protein quantification, 5 μg - 20 μg of total protein were loaded into each well of a Bio-rad ready-made PAGE gel for electrophoresis followed by protein transfer onto a nitrocellulose membrane (10600002 GE Healthcare Life Sciences, Thermo Fisher Scientific). Incubation with a primary antibody was done at 4 °C overnight followed by incubation with a secondary antibody conjugated with horseradish peroxidase (HRP) for 1 hour at room temperature. The signals were developed with the Luminata Forte Western HRP substrate (WBLUF0500 MilliporeSigma) and detected by an Amersham Imager 600 (GE Healthcare Life Sciences). Densitometry was completed with the ImageJ software (NIH)[7, 8].

Immunofluorescence staining (IF). Cultured cells were first fixed with 4% paraformaldehyde for 10 minutes, followed by Triton X-100 permeabilization. After non-specific antibody binding sites were blocked with 10% goat serum, primary antibody was added, and incubation was done at room temperature for 2 hours or at 4°C overnight. After washing with 1x PBS, samples were incubated

with Alexa-Fluor conjugated secondary antibody at room temperature for one hour for signal detection. For IF staining of murine tumor biopsies, 7 μm sections were subjected to fixation and incubation with primary and secondary antibodies as described above. DAPI was used to stain cell nuclei. IF signals were captured under a confocal fluorescence microscope (Zeiss LSM 900 with Airyscan 2, Germany) or the Axio observer with the Zen Blue software (Zeiss, Germany)[7-9].

Analyzing PM Glut1 by Biotinylation. Cells growing in a cell culture plate were incubated with EZ-Link Sulfo-NHS-Biotin (1 mg/mL in PBS, 21217 Thermo Fisher Scientific) for 30 min at 4°C so that proteins at the PM were labeled. After washing off free biotin with 1x PBS, cells were lysed with a lysis buffer (25 mM Tris-HCl pH7.4, 150 mM NaCl, 1% NP40, 1 mM EDTA, and 10% Glycerol)[1, 7-9]. After cell debris was removed by centrifugation, streptavidin agarose beads (S1638 MilliporeSigma) were added into the cell lysate for pulling down biotinylated PM proteins. Beads were then washed with lysis buffer 3 times and precipitated by centrifugation at 10,000 rpm for 2 minutes. PM proteins on the beads were eluted by 2× Loading SDS sample buffer for WB with anti-Glut1 antibody. Data were quantitated by the Image J software (NIH).

Rab11 activity assay. Control and CMTM6 knockdown CRC cells were collected for Rab11 activity assay, which was performed with a Rab11 Activity Assay kit (#83201 NewEast Biosciences, King of Prussia, PA), according to the manufacturer-recommended procedure[4, 10].

Co-immunoprecipitation (coIP). Cells overexpressing CMTM6-HA and Rab11-FLAG were incubated with a crosslink reagent DSP (2 mM in PBS) at 4ºC for 30 minutes followed by cell lysis in a buffer containing 0.5% NP40. 2 μg of anti-HA (12CA5) (11583816001 Roche, MilliporeSigma) or anti-FLAG (F1804 MilliporeSigma), and 25 μL of Protein G Sepharose beads (50% slurry) (17061801 Cytiva, Marlborough, MA) were added into the cell lysates containing equal volume and equal amount of proteins, and incubation was carried out at 4°C overnight. The

beads were precipitated by centrifugation at 10,000 rpm and washed with the lysis buffer. Co-precipitated proteins on the beads were eluted by Laemmle sample buffer (161-0737 BioRad, Hercules, CA) and analyzed by WB with appropriate antibodies[1, 7, 9, 11].

Expression of recombinant protein by baculovirus system. Codon-optimized human Glut1 cDNA was inserted into the pFastBac1 vector (10360014, Thermo Fisher Scientific) at the sites of BamHI and XhoI with a 10× His tag added to its C-terminus. Similarly, human CMTM6 cDNA was inserted into the pFastBac1 vector with a 2x Strep tag added to C-terminus. Recombinant Glut1-His and CMTM6-Strep proteins were then expressed by the pFastBac baculovirus system. Briefly, bacmid DNAs were produced in DH10Bac cells (10361012, Thermo Fisher Scientific) followed by generating baculoviruses with Sf9 insect cells (11496015 Thermo Fisher Scientific). High Five insect cells (B85502 Thermo Fisher Scientific) were transduced by the baculoviruses, and the cells were collected 48 hours later for protein extraction. The lysis buffer contained 25 mM Hepes pH 8.0 and 150 mM NaCl, and the cells were disrupted by a dounce homogenizer on ice. The membrane fraction was obtained by ultracentrifugation at 150,000g for 15 min and solubilized in Hepes buffer (25 mM Hepes pH 8.0 and 150 mM NaCl) containing protease inhibitors (aprotinin at 0.8 mM, pepstatin at 2 mM, leupeptin at 5 mg /ml) and 2% (w/v) n-dodecyl-b-D-maltoside (DDM, Anatrace) at 4ºC for 2 h. After additional ultracentrifugation, the soluble fraction was incubated with nickel affinity resin (30210 Ni-NTA, Qiagen, Hilden, Germany) or Strep-Tactin XT resin (GE29401324, IBA GmBH, MilliporeSigma) at 4 ºC for 1 hour. The resin was rinsed with the wash buffer (His wash buffer: 25 mM Hepes pH 6.0, 150 mM NaCl, 30 mM imidazole, 5% glycerol (w/v), and 0.05% (w/v) DDM (D4641, MilliporeSigma); Strep wash buffer: 25 mM Hepes pH 6.0, 150 mM NaCl, 5% glycerol (w/v), and 0.05% (w/v) DDM) for three times. Lastly,

the proteins were eluted with the wash buffer plus 300 mM imidazole for nickel affinity resin or 50 mM Biotin for Strep-Tactin XT resin.

Glucose uptake assay and flow cytometry. After glucose-starvation for 2 hours, cells were incubated with fluorescent glucose analog 2-[*N*-(7-nitrobenz-2-oxa-1,3-diazol-4-yl) amino]-2-deoxyglucose (2-NBDG 100 μg/mL) (N13195 Thermo Fisher Scientific) for 10 - 15 minutes at 37°C. Cells were then detached from the culture dish by Accutase® solution (A6904 MilliporeSigma) and fixed in 2% paraformaldehyde for 5 minutes. After centrifugation, cells were re-suspended in 200 μL of PBS for flow cytometry for green fluorescence, which was done by a BD LSRFortessaTM X-20 Cell Analyzer and the FlowJo_v10.8.1 software[4].

Agilent Seahorse Glycolysis Stress Test. Cell seeded in a Seahorse XFe96 microplate (103799-100 Agilent Technologies, Inc., Santa Clara, CA) were subjected to Glycolysis Stress Test according to the manufacturer's instructions. In brief, the sensor cartridge was first hydrated overnight followed by incubation in the Agilent Seahorse XF Calibrant (pH = 7.4) (100840-000 Agilent Technologies, Inc.) for 1 hour in a non-CO2 incubator on the day of the assay. Before assay, cell culture medium was replaced with the Seahorse XF DMEM (pH = 7.4) (103575-100 Agilent Technologies, Inc.) and incubated in a non-CO2 incubator for 1 hour at 37 °C as well. Modulating agents, glucose, oligomycin, and 2-DG (103017-100, Agilent Technologies, Inc.) were prepared in Seahorse XF DMEM and loaded into the corresponding injection ports of the sensor cartridge for the assay, which was performed with an Agilent Seahorse XFe96 Analyzer and the Seahorse Wave software. Extracellular acidification rate (ECAR) was recorded real-time with the Seahorse Wave software[4].

RNA sequencing. Control and CMTM6 knockdown HCT116 cells were collected for bulk-cell RNA sequencing. Total RNA was isolated by using a RNeasy Plus Mini Kit (74104 QIAGEN), and the samples were sent to the University of Minnesota Genomic Center for sequencing. Bioinformatics was done with the EdgeR package[2, 7, 9]. Sequencing libraries were created by using the Illumina's Truseq Stranded mRNA Sample Preparation Kit and sequencing was done on HiSeq2500. The Illumina Real Time Analysis (RTA) software and Illumina's CASAVA software 1.8.2 were used for data analysis to generate de-multiplexed FASTQ files. Human genome (hg19) was used as the reference. Data were used as the input for Gene Set Enrichment Analysis (GSEA) (https://www.gsea-msigdb.org/gsea/index.jsp) and annotation was completed by the pathway analysis with the molecular signatures database (C2 pathway)[4]. Volcano plots and heatmaps were generated by using the online software: http://sangerbox.com/home.html and https://build.ngchm.net/NGCHM-web-builder/Select_Matrix.html?v=2.22.0&adv=N (MD Anderson Cancer Center)[4, 9].

Targeted proteomics. Cytokines/chemokines in the conditioned medium (CM) of MC38 cells were analyzed by the Proteome Profiler Mouse XL array kit (ARY028 R&D Systems, Minneapolis, MN, USA). Briefly, 1 mL of CM was mixed with 15 μL of the cocktail of biotinylated antibodies recognizing 111 mouse cytokines/chemokines followed by one hour incubation at room temperature. The antibody/CM mixture were then added onto a nitrocellulose membrane spotted with 111 different antibodies and incubation was done at 4ºC overnight. After washing off unbound antibodies and proteins, streptavidin–HRP and Chemi Reagent were applied for chemiluminescence signal detection. Signals were captured by the ChemiDoc MP Imaging System (Bio-Rad Hercules, CA), and quantitated by the ImageJ software.

Spatial transcriptomics by NanoString GeoMx Digital Spatial Profiler. Tissue sections of MC38 liver metastases were prepared according to the Manual Slide Preparation User Manual (MAN-10150-01) and formalin-fixed sections were sent to the University of Minnesota Genomics Center for probe hybridization, IF, area of interest (AOI) selection, sequencing, and data analysis[4]. Antigen retrieval was done by steaming the slides in 1x Tris-EDTA (pH9.0) followed by Proteinase K digestion at 37ºC for 5 minutes (1 μg/mL) to expose RNA targets. The sections were then incubated with the GeoMx Mouse Whole Transcriptome Atlas Panel (20176 target probes and 210 negative probes) overnight and IF for morphology makers was performed on next day. CAFs were labeled by anti-desmin (ab32362 Abcam), cancer cells were labeled by anti-pan keratin (sc8018, Santa Cruz Technology), and SYTO 13 stained cell nuclei. Immunofluorescence signals were obtained by the GeoMx instrument and areas of interest (AOIs) were selected for data acquisition and analysis. The probe tags cleaved from each AOI were used for library preparation and sequencing, which was done on Illumina NextSeq 2000. FASTQ files were processed to generate counts for each target probe in an AOI. Data were analyzed by the GeoMx DSP Analysis Suite software and subjected to GSEA and heatmap creation, as described in ***RNA sequencing***[4].

Subcutaneous and portal vein tumor injection into mice. All animal studies were approved by the Institutional Animal Care and Use Committee (IACUC) of the University of Minnesota. For subcutaneous injection, liver fibroblasts (0.75×10^6)[1, 7, 8] were mixed with control HCT116 cells or CMTM6 knockdown HCT116 cells (0.75×10^6) respectively followed by subcutaneously co-injecting them into 8-week-old nude mice (553 Charles River, Wilmington, MA). The size of HCT116 nodules was measured by a caliper on different days. Tumor volume was calculated by an equation: tumor volume = $(\text{width})^2 \times \text{length} / 2$[1, 9, 10].

Two months old C57BL6 mice were chosen as the tumor implantation recipients for portal vein injection. Under general anesthesia by isoflurane (2% - 5%), each mouse received 1×10^6 MC38 cells in 100 μL PBS via portal vein injection, as we did previously[1, 2, 9]. Mice received post-operative care for 3 days and they were sacrificed 11 days later.

Statistical analysis. All data are expressed as mean ± SEM, and the Graph-Pad Prism 6 software was used for statistical analysis (GraphPad Software, Inc., La Jolla, CA). For two group comparison, two-tailed Student's *t*-test was performed; for data of more than two groups, ANOVA followed by post hoc tests was performed. $P<0.05$ was considered statistically different.

References for Supplementary Materials and Methods

1. Liu, C. *et al.* IQGAP1 suppresses TbetaRII-mediated myofibroblastic activation and metastatic growth in liver. *J Clin Invest* **123**, 1138-1156 (2013).
2. Dou, C. *et al.* P300 Acetyltransferase Mediates Stiffness-Induced Activation of Hepatic Stellate Cells Into Tumor-Promoting Myofibroblasts. *Gastroenterology* **154**, 2209-2221 e2214 (2018).
3. Wang, Y. *et al.* p300 Acetyltransferase Is a Cytoplasm-to-Nucleus Shuttle for SMAD2/3 and TAZ Nuclear Transport in Transforming Growth Factor beta-Stimulated Hepatic Stellate Cells. *Hepatology* **70**, 1409-1423 (2019).
4. Wang Y, W.X., Bai B, Shaha A, He X, He Y, Ye Z, Shah VH, and Kang N. Targeting Src SH3 domain-mediated glycolysis of HSC suppresses transcriptome, myofibroblastic activation, and colorectal liver metastasis. *Hepatology* https://doi.org/10.1097/HEP.0000000000000763 (2024)
5. Kang, N. *et al.* Focal adhesion assembly in myofibroblasts fosters a microenvironment that promotes tumor growth. *Am J Pathol* **177**, 1888-1900 (2010).

6. Decker, N.K. *et al.* Nitric oxide regulates tumor cell cross-talk with stromal cells in the tumor microenvironment of the liver. *Am J Pathol* **173**, 1002-1012 (2008).
7. Chen, Y. *et al.* Focal Adhesion Kinase Promotes Hepatic Stellate Cell Activation by Regulating Plasma Membrane Localization of TGFbeta Receptor 2. *Hepatol Commun* **4**, 268-283 (2020).
8. Liu, D. *et al.* Protein diaphanous homolog 1 (Diaph1) promotes myofibroblastic activation of hepatic stellate cells by regulating Rab5a activity and TGFbeta receptor endocytosis. *FASEB J* **34**, 7345-7359 (2020).
9. Sun, L. *et al.* PD-L1 promotes myofibroblastic activation of hepatic stellate cells by distinct mechanisms selective for TGF-beta receptor I versus II. *Cell Rep* **38**, 110349 (2022).
10. Tu, K. *et al.* Vasodilator-stimulated phosphoprotein promotes activation of hepatic stellate cells by regulating Rab11-dependent plasma membrane targeting of transforming growth factor beta receptors. *Hepatology* **61**, 361-374 (2015).
11. Liu, C. *et al.* PDGF receptor-alpha promotes TGF-beta signaling in hepatic stellate cells via transcriptional and posttranscriptional regulation of TGF-beta receptors. *Am J Physiol Gastrointest Liver Physiol* **307**, G749-759 (2014).

Supplementary Figure 1

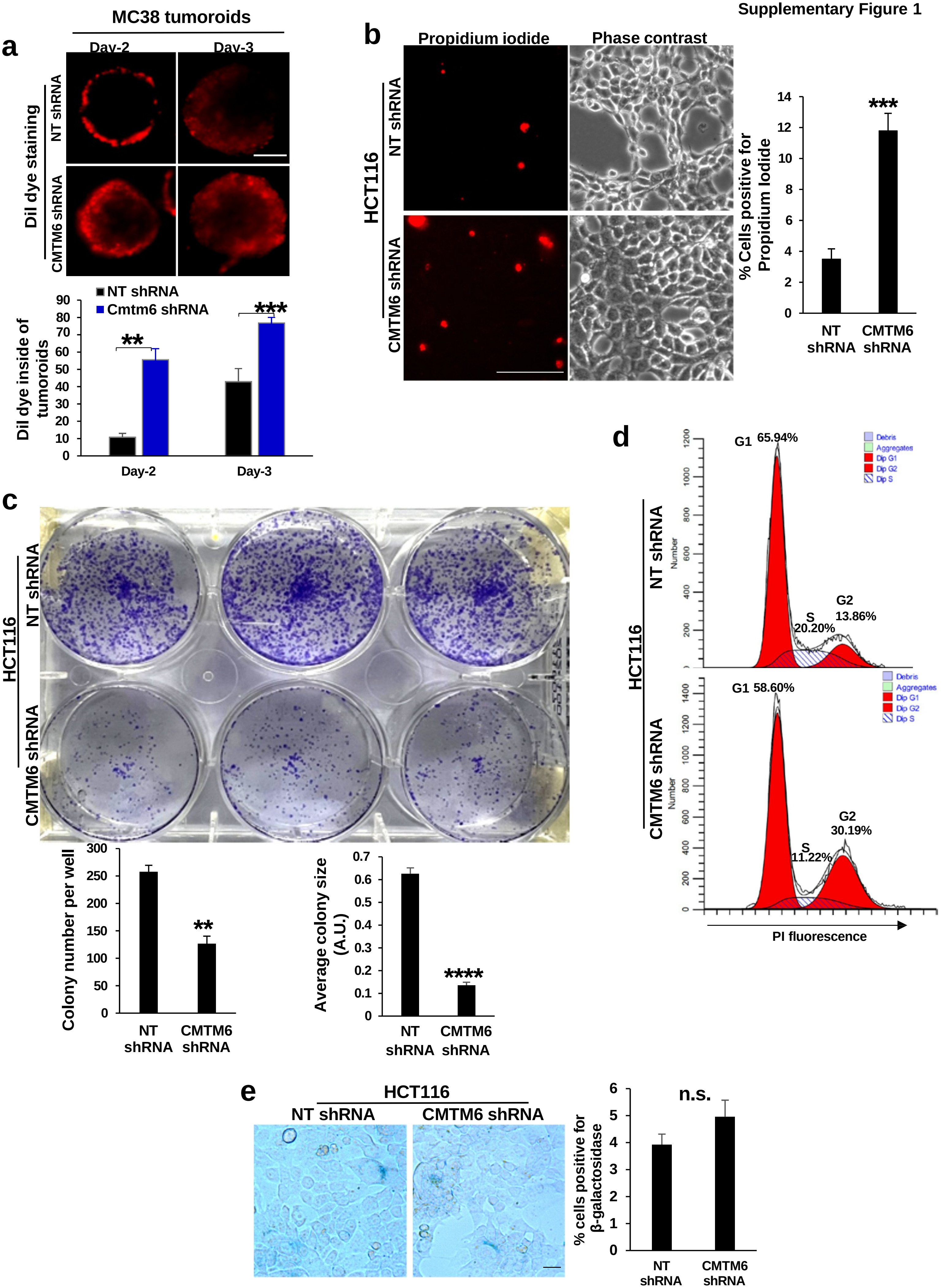

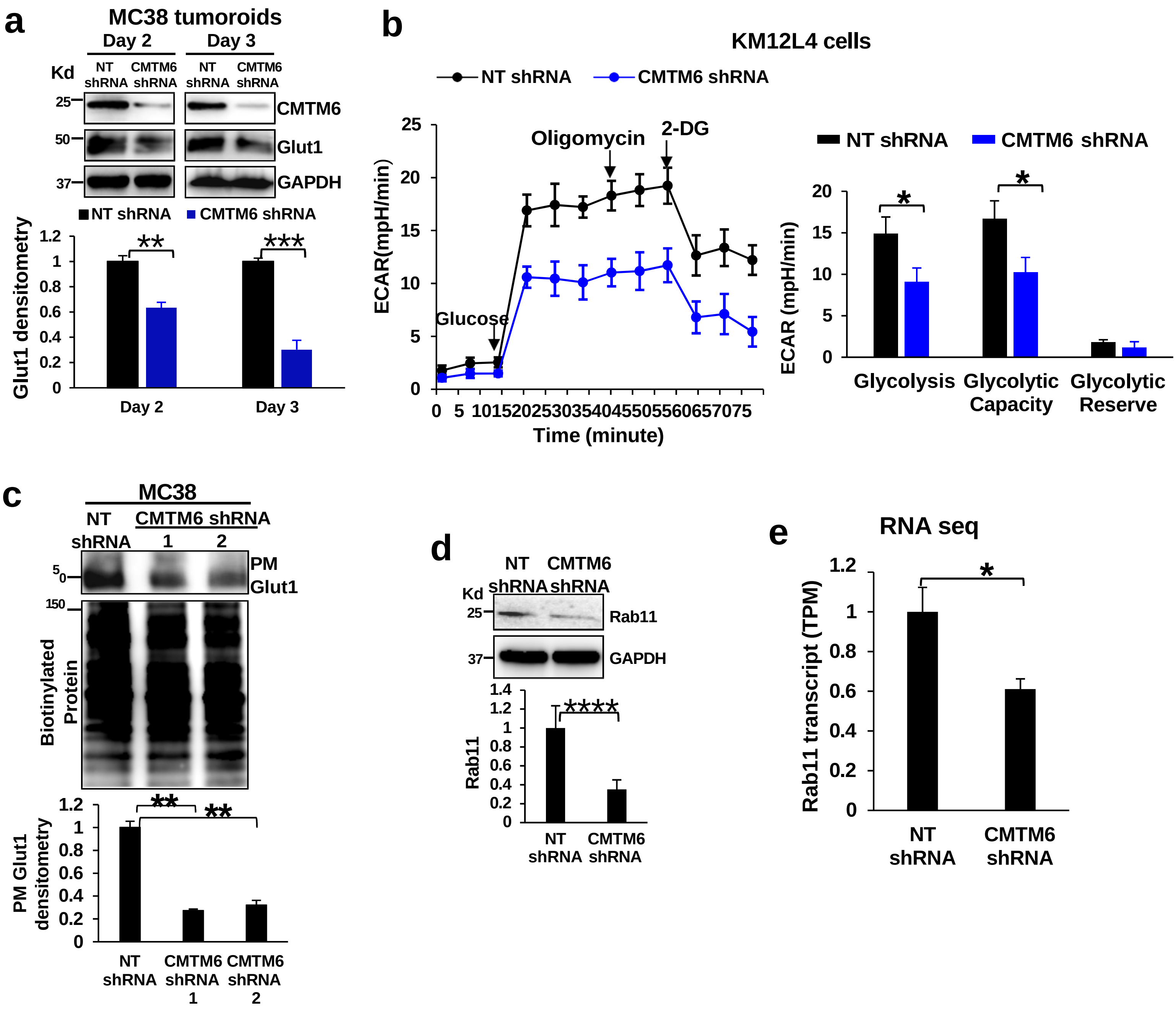
a
MC38 tumoroids
Day 2
Day 3
Kd
NT shRNA
CMTM6 shRNA
25
50
37
CMTM6
Glut1
GAPDH
NT shRNA
CMTM6 shRNA
Glut1 densitometry
**
***
Day 2
Day 3
b
KM12L4 cells
NT shRNA
CMTM6 shRNA
ECAR(mpH/min)
Glucose
Oligomycin
2-DG
Time (minute)
ECAR (mpH/min)
Glycolysis
Glycolytic Capacity
Glycolytic Reserve
c
MC38
NT shRNA
CMTM6 shRNA
1
2
PM Glut1
Biotinylated Protein
150
PM Glut1 densitometry
NT shRNA
CMTM6 shRNA 1
CMTM6 shRNA 2
d
NT shRNA
CMTM6 shRNA
Kd
Rab11
GAPDH
****
e
RNA seq
Rab11 transcript (TPM)
NT shRNA
CMTM6 shRNA

Supplementary Figure 3

A list of the cytokine/chemokine targets of CMTM6

	NT shRNA	CMTM6 shRNA	P-value
**CCL3**	11681.00	0.00	<0.0001
**CCL5**	101844.00	65182.00	<0.0001
**CCL6**	14951.00	0.00	<0.0001
**CCL11**	25340.00	6805.00	<0.0001
**CCL12**	14597.50	0.00	<0.001
**CCL17**	18185.50	0.00	<0.0001
**CCL19**	6954.00	0.00	<0.0001
**CCL22**	18909.50	0.00	<0.0001
**CXCL2**	108681.50	80254.00	<0.001
**CXCL9**	64446.50	25698.00	<0.0001
**CXCL10**	112519.00	88374.00	<0.05
**CXCL11**	8751.50	0.00	<0.0001
**CXCL12**	8977.50	0.00	<0.0001
**IL-1α**	19323.50	0.00	<0.0001
**IL-1β**	16818.50	0.00	<0.0001
**IL-6**	21844.50	6622.50	<0.0001
**IL-17**	15479.50	0.00	<0.0001
**IL-10**	9616.50	0.00	<0.0001
**IL-11**	35283.50	11950.50	<0.0001
**IL-12 p40**	15881.50	0.00	<0.0001
**IL-13**	10975.00	0.00	<0.0001
**IL-15**	16727.50	0.00	<0.0001
**CD14**	18444.50	0.00	<0.0001
**CD40**	28157.50	0.00	<0.0001
**CD160**	9436.00	0.00	<0.0001
**TNF-α**	8478.50	0.00	<0.0001
**VEGF**	121572.00	91289.50	<0.0001

	NT shRNA	CMTM6 shRNA	P-value
**Amphiregulin**	81368.00 SD	42050.50	<0.0001
**Angiopoietin-1**	24489.50	0.00	<0.0001
**Chemerin**	18801.50	0.00	<0.0001
**Chitinase 3-like-1**	11492.00	0.00	<0.0001
**Coagulation Factor III**	57845.50	21776.50	<0.0001
**Endostatin**	22229.50	3658.50	<0.0001
**Fetuin A**	10672.50	0.00	<0.0001
**FGF-21**	27556.50	6940.00	<0.0001
**Flt-3 ligand**	17144.00	0.00	<0.0001
**Gas-6**	10513.50	0.00	<0.0001
**G-CSF**	30908.50	12317.00	<0.0001
**ICAM-1**	22674.00	5268.00	<0.0001
**IGFBP-2**	10432.00	0.00	<0.0001
**IGFBP-3**	19531.00	0.00	<0.0001
**IGFBP-5**	21601.00	5288.00	<0.0001
**LDL R**	119090.00	85962.00	<0.0001
**LIF**	111775.00	86525.00	<0.0001
**Lipocalin-2**	99128.00	70950.50	<0.0001
**LIX**	109838.50	86273.50	<0.0001
**M-CSF**	105964.00	69080.00	<0.0001
**MMP-2**	111638.50	73154.50	<0.0001
**PDGF-BB**	15569.00	0.00	<0.0001
**Pentraxin 2**	24056.00	0.00	<0.0001
**Pentraxin 3**	58024.50	29151.00	<0.0001
**Pref-1**	25931.00	0.00	<0.0001
**Proliferin**	103850.00	66433.00	<0.0001
**CD62E**	15372.50	0.00	<0.0001
**CD62P**	20224.50	0.00	<0.0001
**PAI-1**	101119.50	71982.00	<0.0001
**PEDF**	27174.50	5266.00	<0.0001
**Thrombopoietin**	16764.50	0.00	<0.0001
**TIM-1**	9923.50	0.00	<0.0001
**VCAM-1**	50081.50	24077.00	<0.0001

Supplementary Figure 4

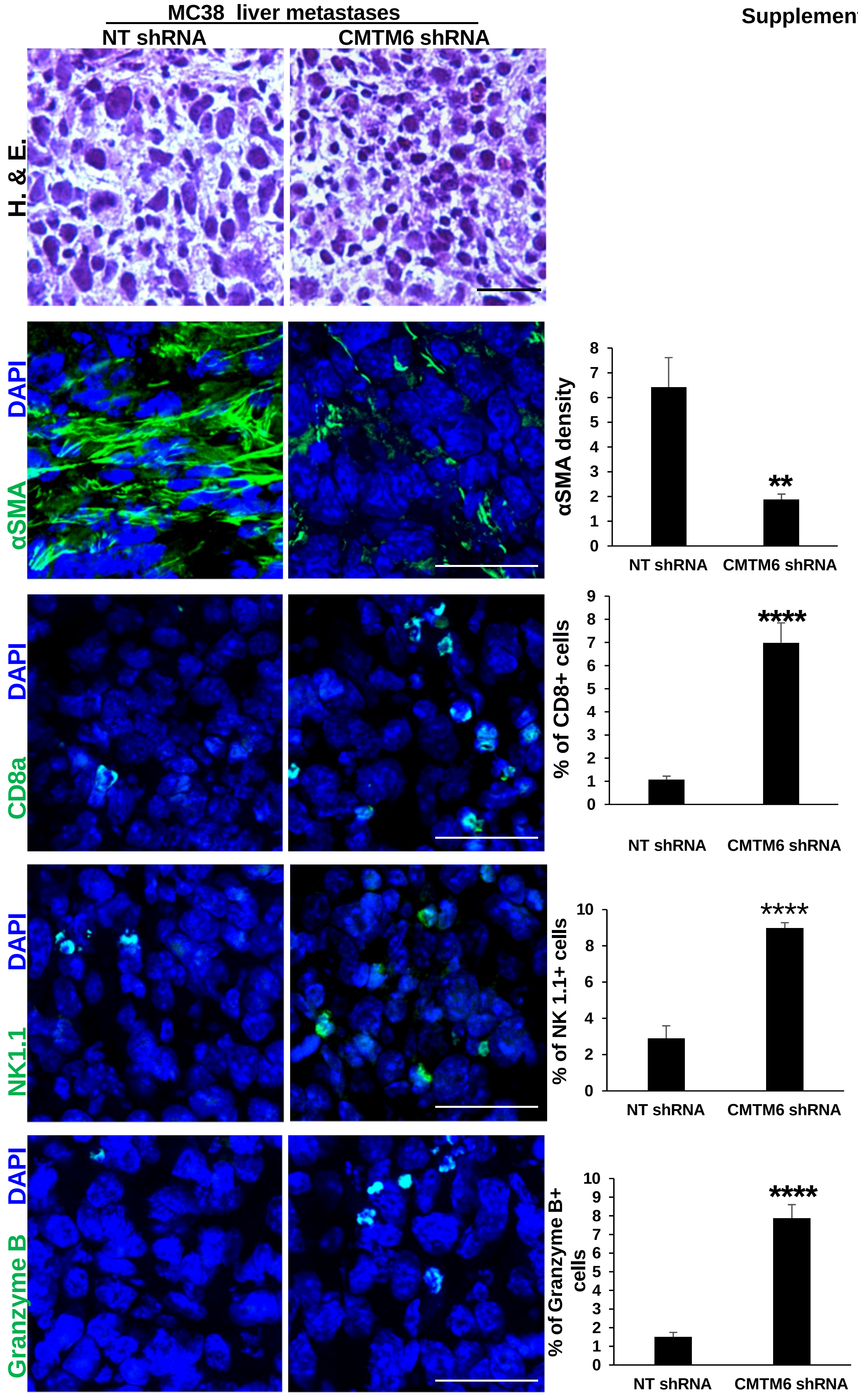

Supplementary Figure 5

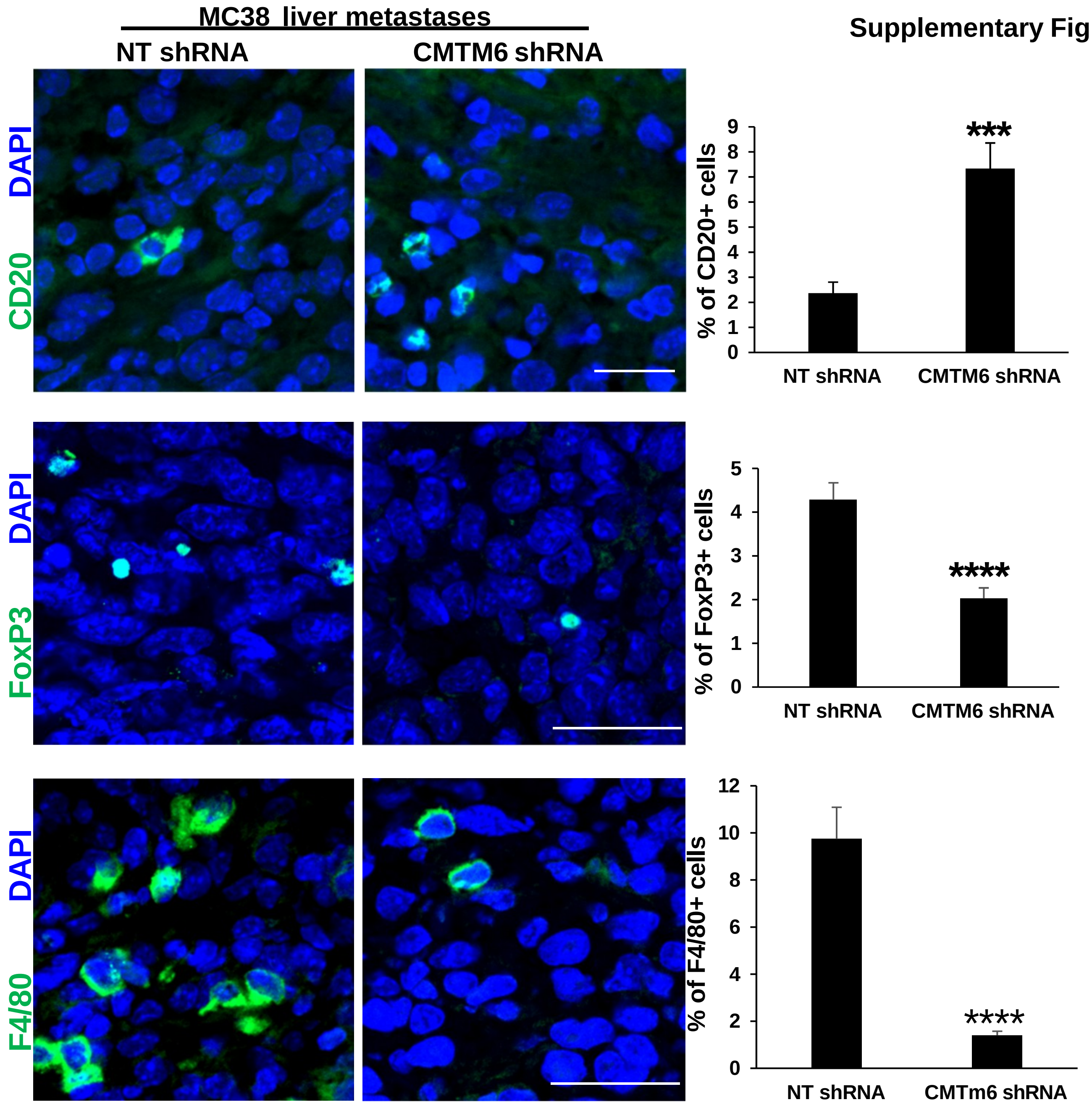

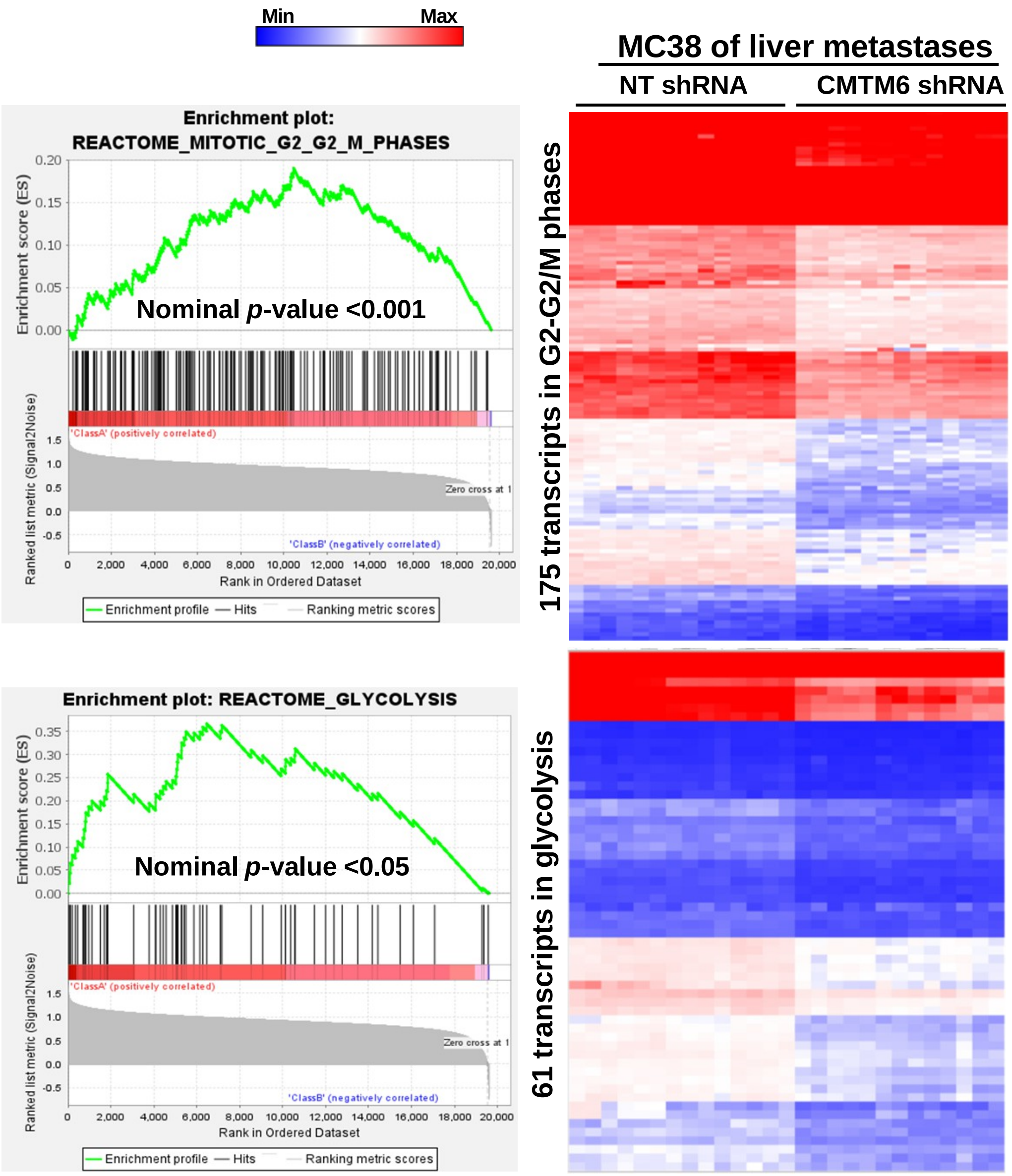
Min
Max
MC38 of liver metastases
NT shRNA
CMTM6 shRNA
Enrichment plot:
REACTOME_MITOTIC_G2_G2_M_PHASES
Enrichment score (ES)
Nominal p-value <0.001
Ranked list metric (Signal2Noise)
'ClassA' (positively correlated)
Zero cross at 1
'ClassB' (negatively correlated)
Rank in Ordered Dataset
Enrichment profile
Hits
Ranking metric scores
175 transcripts in G2-G2/M phases
Enrichment plot: REACTOME_GLYCOLYSIS
Nominal p-value <0.05
61 transcripts in glycolysis

a

The transcripts of cytokines/chemokines affected by CMTM6 knockdown (*P*<0.05)

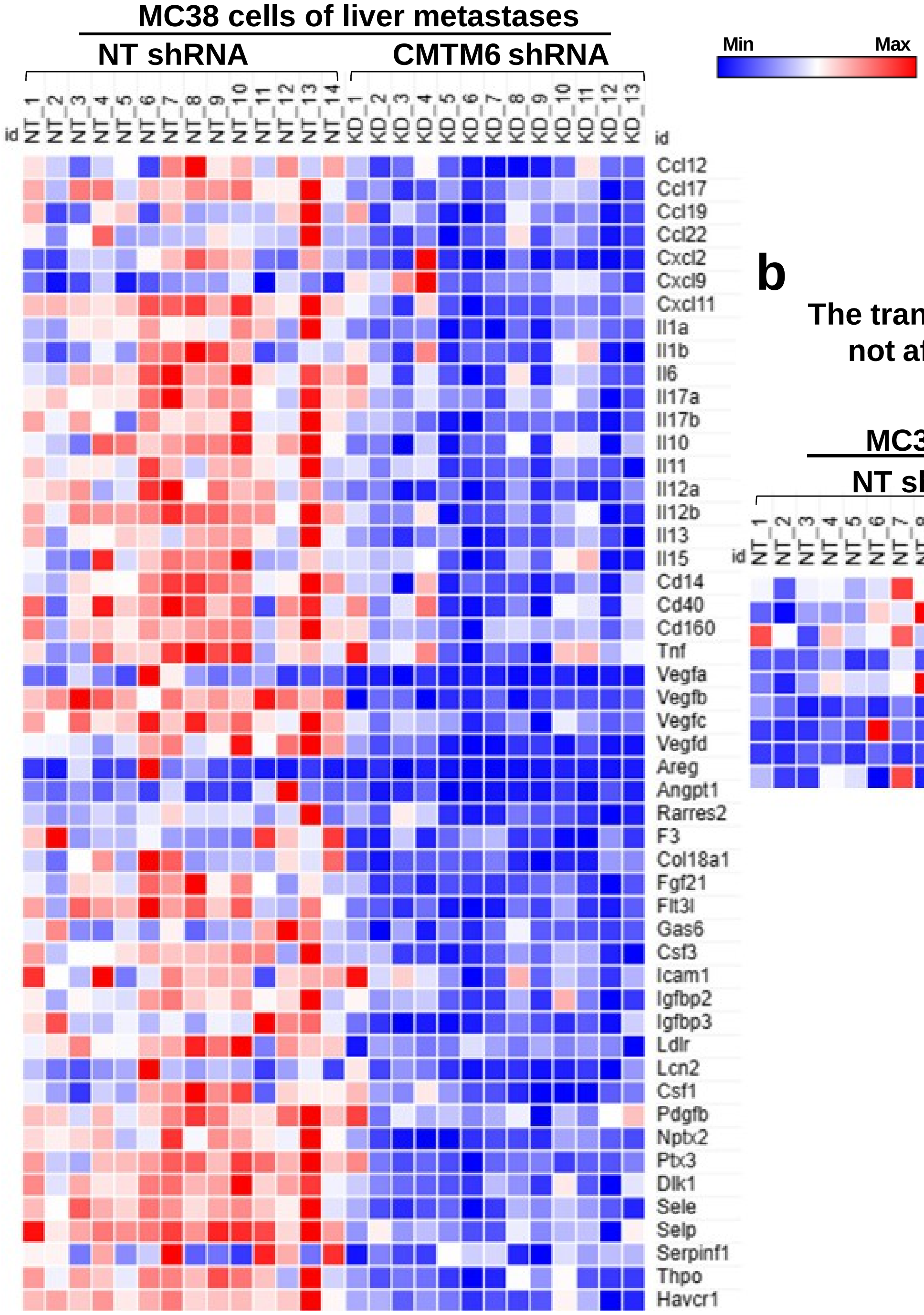

b

The transcripts of cytokines/chemokines not affected by CMTM6 knockdown (*P*>0.05)

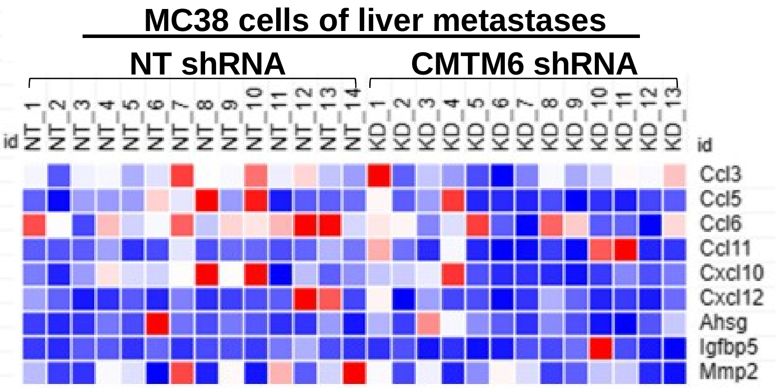

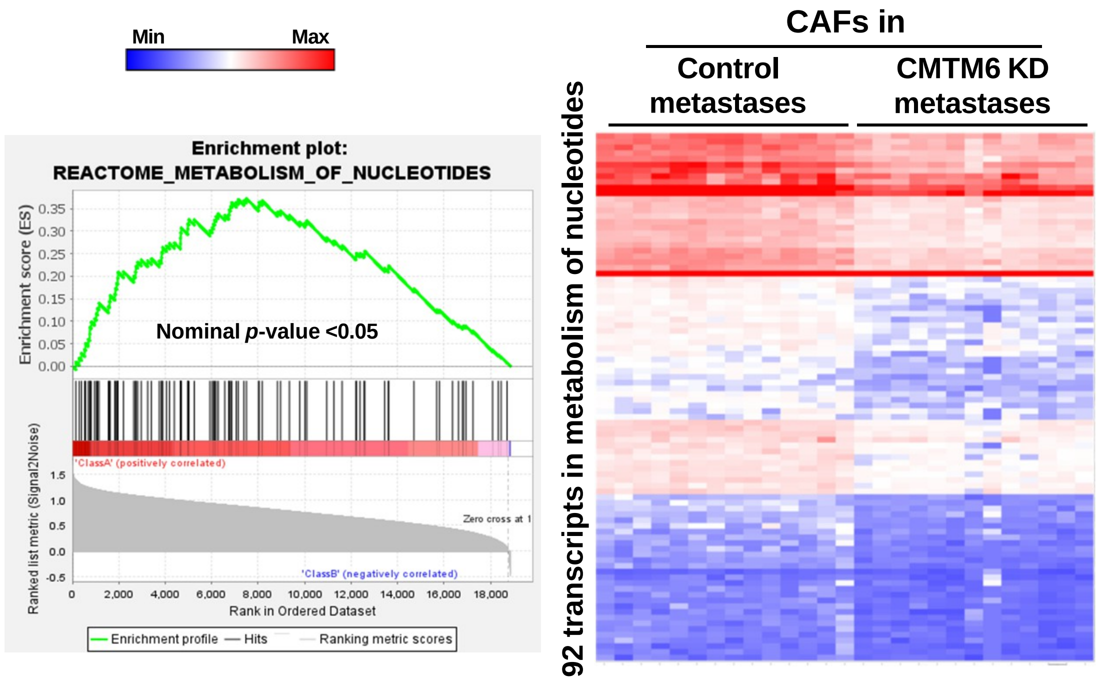
Min
Max
Enrichment plot:
REACTOME_METABOLISM_OF_NUCLEOTIDES
Enrichment score (ES)
Nominal p-value <0.05
'ClassA' (positively correlated)
Zero cross at 1
'ClassB' (negatively correlated)
Ranked list metric (Signal2Noise)
Rank in Ordered Dataset
Enrichment profile
Hits
Ranking metric scores
CAFs in
Control metastases
CMTM6 KD metastases
92 transcripts in metabolism of nucleotides

Supplementary Figure Legends

Supplementary Fig. 1. CMTM6 knockdown influences 3D CRC culture, cell membrane integrity and colony formation. **a.** MC38 tumoroids were subjected to Dil dye staining. CMTM6 knockdown reduced the compactness of the tumoroids. **, *P*<0.01; ***, *P*<0.001 by *ANOVA*, n=3. Bar, 1000 µm. **b.** HCT116 cells were stained with propidium iodide (PI). CMTM6 knockdown significantly increased the number of PI-positive cell. ***, *P*<0.001 by *t*-test, n=5 randomly picked microscopy fields. Bar, 200 µm. **c.** Colony formation assay showed that the number and size of the colonies of HCT116 cells were reduced by CMTM6 knockdown. **, *P*<0.01 by *t*-test, n=3; ****, *P*<0.0001 by *t*-test, n=60 colonies in each group. **d.** Cell cycle analysis of PI-stained cells revealed that the population of cells at the G2 phase of the cell cycle was increased in CMTM6 knockdown HCT116 cells compared to control HCT116 cells. Data are representative of three repeats with similar results. **e.** β-gal staining showed that CMTM6 knockdown did not induce cell senescence of HCT116 cells. n.s., *P*>0.05, n= 8 randomly picked microscopic fields. Bar, 20 µm.

Supplementary Fig. 2. CMTM6 knockdown reduces Glut1 and glycolysis of CRC cells through downregulating Rab11. **a.** WB revealed that Glut1 protein level was reduced by CMTM6 knockdown in 3D MC38 culture. **, *P*<0.01; ***, *P*<0.001 by *ANOVA* n=3. **b.** Seahorse Glycolysis Stress test was performed so as to obtain real-time changes of the extracellular acidification rate (ECAR). Glycolysis was suppressed in CMTM6 knockdown KM12L4 CRC cells compared to control cells. *, *P*<0.05 by *ANOVA*, n=5. **c.** Biotinylation assay revealed that CMTM6 knockdown reduced the level of PM Glut1 of MC38 CRC cells. **, *P*<0.01 by *ANOVA,* n=3. **d.** WB revealed that the total Rab11 protein level was reduced in CMTM6 knockdown HCT116 cells compared to control cells. ****, *P*<0.0001 by *t*-test, n=3. **e.** RNA sequencing revealed that the mRNA level of Rab11 was reduced by CMTM6 knockdown. *, *P*<0.05 *by t*-test, n=3.

Supplementary Fig. 3. CMTM6 controls the release of cytokines/chemokines from MC38. Conditioned medium of control and CMTM6 knockdown MC38 cells were analyzed by the Proteome Profiler Mouse XL Cytokine Array Kit (R&D Systems). CMTM6 knockdown blocked the release of cytokines/chemokines from MC38 cells. Data with *P*<0.05 by *t*-test are shown. n=4.

Supplementary Fig. 4. Targeting MC38 CMTM6 alters the immune suppressive microenvironment of MC38 liver metastases. MC38 liver metastases were subjected to immunofluorescence staining (IF) for αSMA (cancer-associated fibroblast (CAF) marker) and a panel of immune cell markers. Representative IF images demonstrated that the CAF density was reduced whereas that of cytotoxic CD8a+ T cells, NK1.1+ NK/NKT cells, or Granzyme B+ lymphoid cells

was increased in CMTM6 knockdown MC38 liver metastases compared to control liver metastases. **, *P*<0.01; ****, *P*<0.0001 by *t*-test, n=9 - 10 randomly picked microscopic fields. Bar, 20 μm.

Supplementary Fig. 5. Targeting MC38 CMTM6 alters the immune suppressive microenvironment of MC38 liver metastases. Representative IF images demonstrated that the cell density of CD20+ B cells was increased whereas that of FoxP3+ T cell or F4/80+ macrophages was reduced in CMTM6 knockdown MC38 liver metastases compared to control liver metastases. ***, *P*<0.001; ****, *P*<0.0001 by *t*-test, n=9 - 10 randomly picked microscopic fields. Bar, 20 μm.

Supplementary Fig. 6. Spatialomics revealing global transcriptomic changes in CMTM6 knockdown liver metastases. 14 areas of interest (AOIs) in CMTM6 knockdown MC38 liver metastases and 13 AOIs in control liver metastases were subjected to Spatial transcriptomics study. Two gene sets, G2 G2/M phases and glycolysis, with their transcripts affected in CMTM6 knockdown MC38 cells are shown. Transcript enrichment is shown by an enrichment plot (left) and the transcript levels are shown by a heatmap (right). n=14,13. The bar represents the minimum (blue) to the maximum expression level (red).

Supplementary Fig. 7. The transcripts of the cytokine/chemokine targets of CMTM6 in MC38 liver metastases. **a.** A heatmap, based on Spatial transcriptomics, revealing that the transcripts of 50 cytokine/chemokine targets of CMTM6 were reduced in MC38 cells of CMTM6 knockdown liver metastases. *P*<0.05 by *t*-test, n=14,13. **b.** A heatmap showing that the transcripts of 9 cytokine/chemokine targets of CMTM6 were not reduced in MC38 cells of CMTM6 knockdown liver metastases. *P*>0.05 by *t*-test, n=14,13. The bar represents the minimum (blue) to the maximum expression level (red).

Supplementary Fig. 8. Spatialomics revealing global transcriptomic changes in the CAFs of CMTM6 knockdown MC38 liver metastases. The CAFs in each AOI were also subjected to Spatial transcriptomics study. A CAF gene set related to the metabolism of nucleotide was affected by targeting MC38 CMTM6. Transcript enrichment is shown by an enrichment plot (left) and transcript levels are shown by a heatmap (right). n=14,13. The bar represents the minimum (blue) to the maximum expression level (red). KD, knockdown.
